# Microbiota dysbiosis influences immune system and muscle pathophysiology of dystrophin deficient mice

**DOI:** 10.1038/s44321-026-00445-1

**Published:** 2026-06-03

**Authors:** Andrea Farini, Luana Tripodi, Chiara Villa, Francesco Strati, Amanda Facoetti, Guido Baselli, Jacopo Troisi, Annamaria Landolfi, Caterina Lonati, Davide Molinaro, Michelle Wintzinger, Stefano Gatti, Barbara Cassani, Flavio Caprioli, Federica Facciotti, Mattia Quattrocelli, Yvan Torrente

**Affiliations:** 1 Stem Cell Laboratory, Department of Pathophysiology and Transplantation, Università degli Studi di Milano, Unit of Neurology, Fondazione IRCCS Ca’ Granda Ospedale Maggiore Policlinico, Centro Dino Ferrari, Milan, Italy; 2 Mucosal Immunology Lab, Department of Experimental Oncology, IEO-European Institute of Oncology, Milano, Italy; 3 Humanitas University, Milan, Italy; 4 Translational Medicine – Department of Transfusion Medicine and Hematology, Fondazione IRCCS Ca’ Granda Ospedale Maggiore Policlinico, Milan, Italy; 5 Department of Medicine, Surgery and Dentistry, Scuola Medica Salernitana, University of Salerno, Baronissi, Italy; 6 Theoreo srl, Spinoff Company of the University of Salerno, Montecorvino Pugliano, Italy; 7 Center for Surgical Research, Fondazione IRCCS Ca’ Granda, Ospedale Maggiore Policlinico, Milan, Italy; 8 Molecular Cardiovascular Biology Division, Heart Institute, Cincinnati Children’s Hospital Medical Center, and Department of Pediatrics, University of Cincinnati College of Medicine, Cincinnati, OH USA; 9 Department of Medical Biotechnologies and Translational Medicine, Università Degli Studi di Milano, Milan, Italy; 10 Humanitas Clinical and Research Center IRCCS, Milan, Italy; 11 Department of Pathophysiology and Transplantation, Università degli Studi di Milano, Unit of Gastroenterology and Endoscopy, Fondazione IRCCS Ca’ Granda, Ospedale Policlinico di Milano, Milano, Italy; 12 Present Address: SciLifeLab, Department of Microbiology, Tumor and Cell Biology, Karolinska Institutet, Solna, Sweden

**Keywords:** Digestive System, Microbiology, Virology & Host Pathogen Interaction, Musculoskeletal System

## Abstract

Duchenne muscular dystrophy (DMD) is a progressive, severe muscle-wasting disease caused by mutations in *DMD*, encoding dystrophin, that leads to loss of muscle function with cardiac/respiratory failure and premature death. Since dystrophic muscles are sensed by infiltrating inflammatory cells, and gut microbial communities can cause immune dysregulation and metabolic syndrome, we sought to investigate whether intestinal bacteria support the muscle immune response in the mdx dystrophic murine model. We highlighted a strong correlation between DMD disease features and the relative abundance of *Prevotella*. Furthermore, the absence of gut microbes through the generation of mdx germ-free animal model, as well as modulation of the microbial community structure by antibiotic treatment, influenced muscle immunity and fibrosis. Intestinal colonization of mdx mice with eubiotic microbiota was sufficient to reduce inflammation and improve muscle pathology and function. This work identifies a potential role for the gut microbiota in the pathogenesis of DMD.

## Synopsis

The susceptibility of DMD patients to inflammatory events cannot solely be explained by skeletal muscle genetic defects, but rather favours a new paradigm linking development of chronic inflammation with a strict regulation between epigenetics factors and degenerative environment.

- Gut microbiota specific alterations (dysbiosis) correlates with the dystrophic pathology in mdx mice, influencing muscle immunity and fibrosis.
- Dysbiotic mdx microbiota induces a decreased innate immune response and altered muscle metabolism.
- The study of the dysregulated immune system-microbiota axis in mdx mice highlights the importance of microbiota as potential target for therapeutic interventions.

## Abstract (web summary)

The susceptibility of DMD patients to inflammatory events cannot solely be explained by skeletal muscle genetic defects, but rather favours a new paradigm linking development of chronic inflammation with a strict regulation between epigenetics factors and degenerative environment.

## Graphical abstract

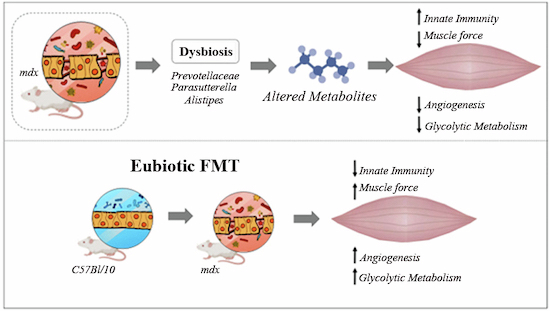

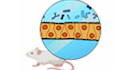

The paper explained **Problem**

Recent studies indicate that chronic inflammation partly depends on the tissue-environment interface. In the gastrointestinal mucosa, immune cells such as antigen-presenting cells and lymphocytes drive innate and adaptive responses. Their dysregulation triggers inflammatory events that are hallmarks of Duchenne muscular dystrophy (DMD). Although gut microbiota likely influences muscle metabolism and physiology, the molecular mediators of the gut-muscle axis remain unidentified. Increased intestinal permeability releases molecules that promote muscle wasting. Gut microbiota produce metabolites accessible to tissues, modulate systemic immunity, and affect distant sites via the circulation. Moreover, bacteria can translocate into the bloodstream through a leaky gut barrier and contribute to muscle pathology. In muscular dystrophies, dystrophin deficiency severely disrupts intestinal homeostasis, leading to gastrointestinal symptoms that often precede classic DMD musculoskeletal features. Despite evidence that microbiota modulation could exacerbate DMD inflammation, gastrointestinal dysfunction in this disease remains understudied.

**Results**

We first observed reduced microbial richness in 3-month-old mdx mice (a DMD model) compared to age-matched wild-type C57BL/6 mice, with enrichment in *Prevotella* genus and distinct metabolic profiles. Using mdx models of varying disease severity, we established a correlation between gut inflammation and muscle damage. To test dysbiosis’s role in muscle wasting, we generated germ-free dystrophic mice (GFmdx). Our analyses revealed mucosal, immunological, and microbial alterations driving intestinal inflammation in dystrophic mice. Notably, microbiota depletion in 3-month-old mdx mice dampened innate immune responses but altered muscle metabolism and function. Fecal microbiota transplantation from mdx mice into wild-type recipients confirmed that dysbiosis promotes inflammation in the intestine, spleen, and muscle, inversely correlating with muscle function.

**Impact**

Nutraceuticals and dietary metabolites show promise as adjunct therapies for DMD, alongside immunomodulators. Identifying inflammation-triggering bacteria could enable targeted microbial manipulation for therapeutic benefit. Corticosteroids remain the standard treatment, extending ambulation and preserving muscle function, but they cause severe side effects. Immunomodulatory nutraceuticals could improve the quality of life and reduce hospitalization costs linked to corticosteroid complications.

## Introduction

Duchenne muscular dystrophy (DMD) is an X-linked disease caused by mutations in the *DMD* gene and loss of the dystrophin protein, leading to myofiber membrane fragility and necrosis with weakness and contractures. Affected DMD boys typically die in their second or third decade of life due to either respiratory failure or cardiomyopathy (Emery, 2002). Although the primary defects rely on skeletal muscle structure, a multitude of secondary defects exist involving deregulated metabolic and inflammatory pathways. Immune cell infiltration into skeletal muscle is, indeed, a typical feature of DMD pathophysiology and strongly associated with disease severity (Evans et al, 2009). In the dystrophic dystrophin-deficient mdx murine model, we recently found the presence of activated T lymphocytes and the overexpression of immunoproteasome (IP), an enzymatic complex that cleaves peptides to produce epitopes for antigen presentation to T lymphocytes. We have demonstrated that IP inhibition improved dystrophic muscle functions by reducing the number of both circulating and infiltrating activated T cells, confirming a pathogenic role of immune cells (Farini et al, 2016). Dystrophic muscle features were also improved by depletion of B cells and T cells in immunodeficient dystrophinopathic (SCID/mdx) and dysferlinopathic (SCID/BlAJ) murine models (Farini et al, 2012). So far, immunosuppressive drugs, such as glucocorticoids, are the only effective therapies to delay the onset and control symptoms (Zhang et al, 2022a), ameliorating ambulation and muscle function, but their use in patients is still limited by serious side effects. In this scenario, the individual susceptibility to inflammatory events cannot be simply explained with the genetic defects of skeletal muscles; rather, there is a new emerging paradigm explaining the development of chronic inflammation that comprehends a strict regulation of epigenetics factors, genetic components, and the environment. In particular, environmental intrinsic (as innate and adaptive immunity) and extrinsic (as nutrition) mechanisms are connected to each other in a well-defined temporal and spatial way, whose dysfunctions are the main causes of chronic inflammatory conditions (Mollaei et al, 2020). The tissue-environment interface is a preferential site critically involved in triggering mechanisms of chronic inflammation, especially in the mucosa of the gastrointestinal tract, where commensal microorganisms forming the so-called microbiota provide nutrients by digestion of dietary components, modulate the development of the mucosal immune system, and protect from pathogens (Yan et al, 2016). Thus, alterations of gut microbial communities (dysbiosis) can cause immune dysregulation and metabolic syndrome, contributing to a multitude of diseases of different aetiologies (Zheng et al, 2020). The commensal population that constitutes the microbiota is extremely variable among individuals, and its composition is dependent on the immune responses that are mediated in the gut and on host genotypes/phenotypes (Spor et al, 2011). Accordingly, the intestinal homeostasis is maintained through the mutualistic interactions between the microbiota and intestinal immune cells: dysfunctions cause serious problems, including a chronic inflammatory state (Kabat et al, 2016). In some way, gut microbiota modulation can also alter the regulatory molecules secreted by skeletal muscles and adipose tissues—myokines and adipokines—whose function is strictly dependent on the production of short-chain fatty acids (SCFAs) and branched-chain amino acids (Suriano et al, 2020). In muscle tissue, dysbiosis interferes with the proper development of muscle progenitor cells, likely through reactive oxygen species generation and antioxidant genes (Tidball, 2017), and with endothelial cell function (Amedei and Morbidelli, 2019). The latter has already been confirmed by the occurrence of vascular development dysfunctions in pathogen-free mice, possibly dependent on defects in nitric oxide synthase activity and in the expression of vessel inflammatory genes that were reversed by the restoration of normal gut flora (Joe et al, 2020).

Duchenne muscular dystrophy patients present alterations of gastrointestinal motility and suffer from constipation, pseudo-obstruction, and acute dilatation. Although no attention was paid to investigate these processes, smooth muscle fibrosis was observed throughout the gastrointestinal tract (Mule et al, 2010). Mdx mice shared impairments in intestinal contractility, linked to important abnormalities of the mucosal epithelial morphology (wider villi, reduced muscular and submucosa layer) normally associated with an inflammatory state (Feder et al, 2018), and especially to nitric oxide (NO) production (Mule et al, 2001). In addition, it was demonstrated that L-arginine dietary supplementation improved colonic motility and increased NO signaling, ameliorating the pathological phenotype of mdx mice (Swiderski et al, 2020). These evidences confirmed the involvement of a muscle-gut axis-mediated pathway that contributes to jeopardize the pathophysiology of DMD (Alves et al, 2014). Modulation of microbiota is also responsible for modifications of immunological and inflammatory features in organs distant from the gut. For instance, experiments of gnotobiology have shown that inhibition of microbiota function diminished the development of arthritis and autoimmune encephalomyelitis in murine models, whereas colonization of germ-free mice with specific bacterial strains modified the expression of circulating monocytes, Th17 T lymphocytes, and B lymphocytes (Belkaid et al, 2013; Yang et al, 2023). Likely, inflammatory molecules derived from microbiota leak from the disrupted gut barrier of the mdx mice, activate inflammatory cells, and circulate through the blood towards muscles, where they modulate the immune system, worsening the dystrophic phenotype.

Thus, it is possible to envision a connection between gut microbiota, immunity, and muscle homeostasis, but the molecular details of this cascade of events in muscular dystrophy are still elusive. Here, we demonstrate dysbiosis in mdx mice that is associated with alterations of the peripheral and local mdx immune landscape and muscle integrity. Treatment with broad-spectrum antibiotics depleting the gut microbiota in 3-month-old (3 m) mdx mice determined reduced muscle inflammation and enhanced fatty acid oxidation with consequent shift in fiber type toward an oxidative phenotype associated with muscle wasting. We also show that germ-free mdx (GFmdx) mice were impaired in muscle function except for reduced fibrosis and absence of chronic muscle inflammation. Furthermore, intestinal colonization of mdx mice with eubiotic microbiota was sufficient to reduce inflammation, improving muscle pathology and function. Our work provides new insights into DMD pathogenesis by highlighting the role of intestinal microbiota in shaping the muscular inflammatory response and conferring a distinct susceptibility to dystrophic muscle disease. From a therapeutic perspective, our results allow the identification of intestinal microorganisms, microbial products, and metabolites as potential targets to tailor innovative therapeutic strategies for the treatment of DMD patients.

## Results

### Altered gut microbiota composition in mdx mice

**Figure 1:**
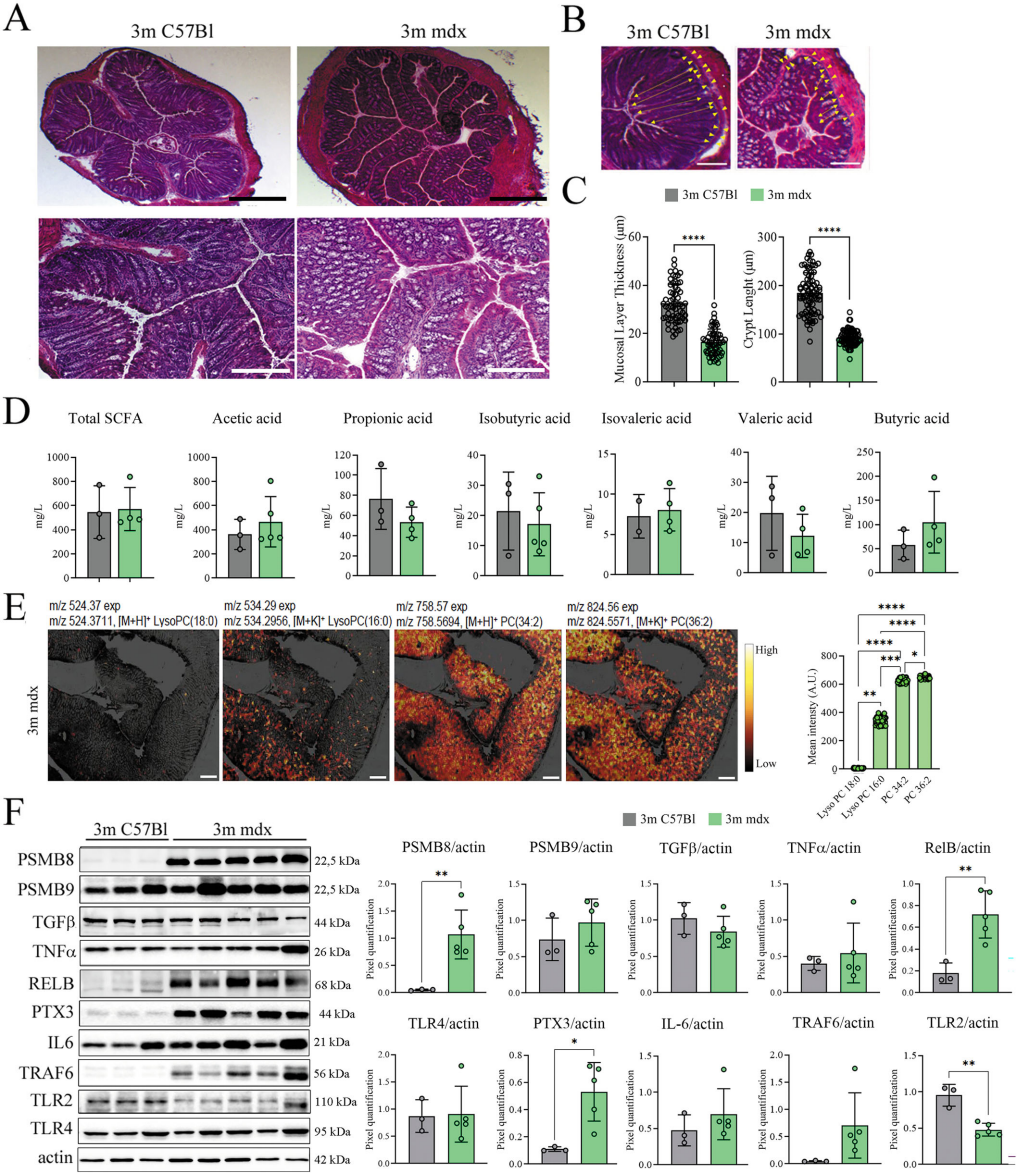
Colon characterization of 3 m mdx mice. (**A**) Representative images of H&E staining of colon from 3 m C57Bl (*n* = 4) and mdx (*n* = 4) mice. High magnification (scale bar: 20 μm) and low magnification (scale bar: 200 μm). (**B**) Mucus layer, area between yellow dash lines; crypt length, yellow-headed arrow. Scale bar: 100 μm. (**C**) Mucus layer thickness and crypt length were quantified for *n* = 4 mice per group (with pooled samples of *n* = 60 for mucus layer thickness and *n* = 80 for crypt length). (**D**) Short-chain fatty acid fecal quantification of 3 m C57Bl (*n* = 3) and mdx (*n* = 4–5) mice. (**E**) Colon images captured with the iMScope TRIO described an altered pattern of expression of different Phosphatidylcholines (PC) and Lysophosphatidylcholines (LysoPC) (as indicated by m/z values) in 3 m mdx mice (*n* = 3). Scale bar: 50 μm. For each lipid, the mean intensities measured at 12 positions throughout colon images are shown on the right side, where bars are mean ± SEM (*n*  =  3). (**F**) Cropped images of representative WB showing the expression of proteins involved in inflammation and fibrosis in colon tissues of 3 m C57Bl (*n* = 3) and 3 m mdx (*n* = 5). Densitometric analyses of protein expression are shown as a ratio to actin. Data are presented as mean ± SD (*P < 0.05; **P < 0.01, ***P < 0.001; Student *t* test). Source data are available online for this figure.

Full-length dystrophin is predominantly expressed by skeletal muscles, but dystrophic patients present impairment of gastrointestinal functions, altered motility, and histological evidence for smooth muscle fibrosis throughout the gastrointestinal tract (Mule et al, 2010). Mdx mice shared impairments in the intestinal contractility, mainly due to NO dysfunctions (Baccari et al, 2000), increased calcium influx, and deregulated tachinergic NK2 receptors (Mule and Serio, 2001). Slow fecal output and transit time of fecal material revealed motor disturbance in mdx mice, highlighting a delay in the propulsion (Mule et al, 2010). We found alterations of histological structures in the colon of 3 m mdx mice, mainly consisting of epithelial atrophy, shorter villi, and thinner muscular and submucosal layers (Fig. 1A–C). Total SCFA content in the stool isolated from colon, which contributes to immune regulation and anti-inflammatory effects (Correa-Oliveira et al, 2016), was found to be similar between 3 m C57Bl wild-type (WT) and 3 m mdx mice (Fig. 1D). However, the imaging mass spectrometry of lipids in mdx small intestine tissues demonstrated an enrichment of phosphatidylcholines (PC: 34:2; PC 36:2) and PC cleavage byproducts as lysophosphatidylcholines (LysoPC: 16:0) (Fig. 1E). PC and LysoPC activate multiple signaling pathways that are involved in oxidative stress and inflammatory responses triggered through Toll-like receptors (TLRs) (Carneiro et al, 2013) leading to increased release of cytokines—i.e., interleukin (IL)-1β, IL-6, and tumor necrosis factor-α (TNFα) (Sato et al, 2014)—and activation of lymphocytes (Huang et al, 1999) and pro-inflammatory M1 macrophages (Yang et al, 2005). Compared with WT, the mdx intestinal tissues showed a trend for increased pro-inflammatory IL-6 and TNFα cytokines and TNF receptor-associated factor 6 (TRAF6) inflammatory mediator (Fig. 1F). Accordingly, significant upregulation of mediators of innate immunity, as proteasome subunit beta type-8 (PMSB8), pentraxin-3 (PTX-3) and v-rel Reticuloendotheliosis viral oncogene homolog B (RelB), was observed in mdx vs WT intestine tissues (Fig. 1F). Modifications of neither transforming growth factor (TGF)-β1 nor TLR4 were found (Fig. 1F). Interestingly, we determined that TLR2—which is involved in maintenance of tight junction integrity and regulation of gut chronic inflammation (Cario, 2008; Rakoff-Nahoum et al, 2004)—was significantly downregulated in mdx intestine (Fig. 1F).

**Figure 2:**
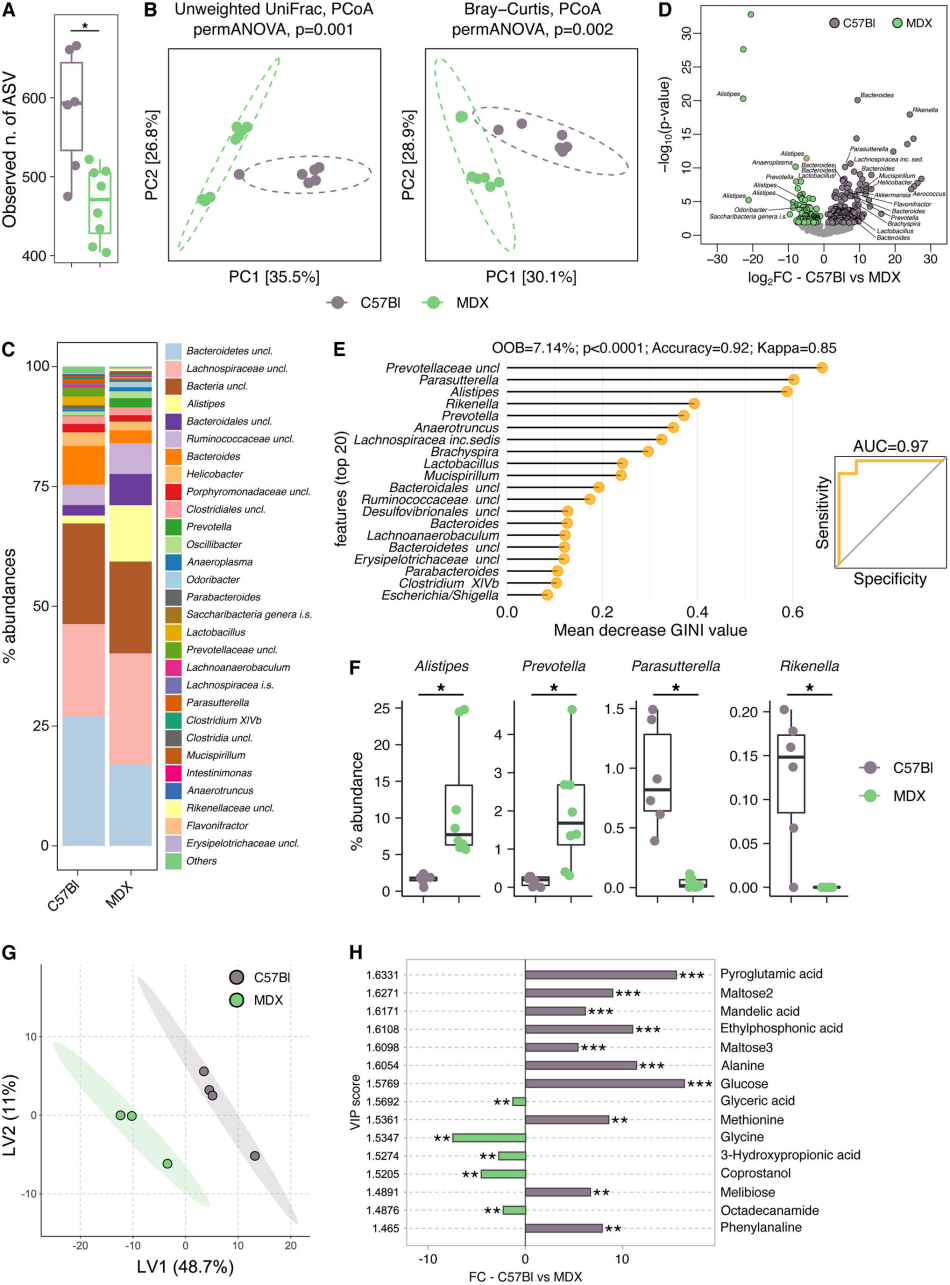
Microbiome analysis of dystrophic mdx mice. (**A**) Observed number of enriched ASVs in 3 m C57Bl (*n* = 6) (maximum: 666; median: 583.67; minimum: 475) and 3 m mdx (*n* = 8) (maximum: 522; median: 465.625; minimum: 404). Data are presented as the exact number of ASVs (*P < 0.05; Student’s *t* test). (**B**) PCA of beta-diversity of 3 m C57Bl (*n* = 6) and 3 m mdx (*n* = 7) as measured by Unweighted UniFrac distance and Bray–Curtis dissimilarity. (**C**) Mean relative abundance at genus level among groups. All genera with relative abundance < 0.1% are reported together and labeled as “others”. (**D**) Volcano plots of 3 m C57Bl (*n* = 6) and 3 m mdx (*n* = 7) showing the significantly enriched bacterial ASVs (with *P* < 0.05) by the DEseq2 analysis. The names of the significantly enriched bacterial ASVs classified to the genus level and *P* < 0.005 are reported. All *P* values were false discovery rate-corrected. (**E**) Random forest analysis. The top 20 bacterial genera with the highest discriminatory power, sorted by mean decrease GINI value, are shown. (**F**) Relative abundance of different genera in 3 m C57Bl (*n* = 6) and 3 m mdx (*n* = 7). *Prevotella*: 3 m C57Bl: maximum: 0.28438734; median: 0.162312199; minimum: 0. 3 m mdx: maximum: 4.650449086; median: 1.928565583; minimum: 0.308938765. *Alistipes*: 3 m C57Bl: maximum: 2.381488226; median: 1.622780995; minimum: 0.52990159. 3 m mdx: maximum: 24.79693926; median: 11.74146327; minimum: 5.71434417. *Parasutterella*: 3 m C57Bl: maximum: 1.491499069; median: 0.923747366; minimum: 0.390776848. 3 m mdx: maximum: 0.114573317; median: 0.036571394; minimum: 0.002045952. *Rikenella*: 3 m C57Bl: maximum: 0.202549256; median: 0.124121093; minimum: 0.067516419. 3 m mdx: maximum: 0; median: 0; minimum: 0. *P < 0.05; Student’s *t* test. (**G**) Multidimensional scaling analysis of small intestinal metabolomic profiles from 3 m C57Bl (*n* = 4) and 3 m mdx (*n* = 3) mice calculated by samples’ distance similarities (Bray–Curtis) with the most discriminatory metabolites (top VIP-score) identified. (**H**) Concentration of the significantly different metabolites isolated from the small intestinal content of 3 m C57Bl (*n* = 4) and 3 m mdx (*n* = 3) mice. *P < 0.05, **P < 0.01, and ***P < 0.001; Wilcoxon rank-sum test. Source data are available online for this figure.

To verify the intestinal microbial community structure, we performed a metataxonomic analysis. The analysis of gut microbiota alpha-diversity showed a significant reduction of microbial richness in 3 m mdx compared to age-matched C57Bl animals (Fig. 2A; FDR-corrected *P* < 0.05, pairwise comparisons using the Wilcoxon rank-sum test), suggesting that mdx mice are characterized by a dysbiotic microbiota. The microbial community structures among groups were significantly different as measured by beta-diversity of Unweighted UniFrac distances and Bray–Curtis dissimilarity (Fig. 2B; Dataset EV1; PERMANOVA *P* < 0.05). An in-depth analysis of the gut microbiota (Fig. 2C,D) showed the enrichment of different amplicon sequence variants (ASV) belonging to the genera *Alistipes* and *Prevotella*, among others, in 3 m mdx compared with C57Bl mice. Notably, LysoPC has been associated with the abundance of the genus *Alistipes* (Li et al, 2022; Zhang et al, 2022b). By using a Random Forest classifier, we further observed that the gut microbiota was able to classify samples according to status (OOB = 18.2%; *P* < 0.0001; Accuracy = 0.81; Kappa = 0.725) and that the genus *Prevotella* was the most important, fully classified, feature to categorize samples according to status (Fig. 2E). According to these data, *Prevotella* was among the taxa whose abundance was significantly higher in 3 m mdx.

**Figure 3:**
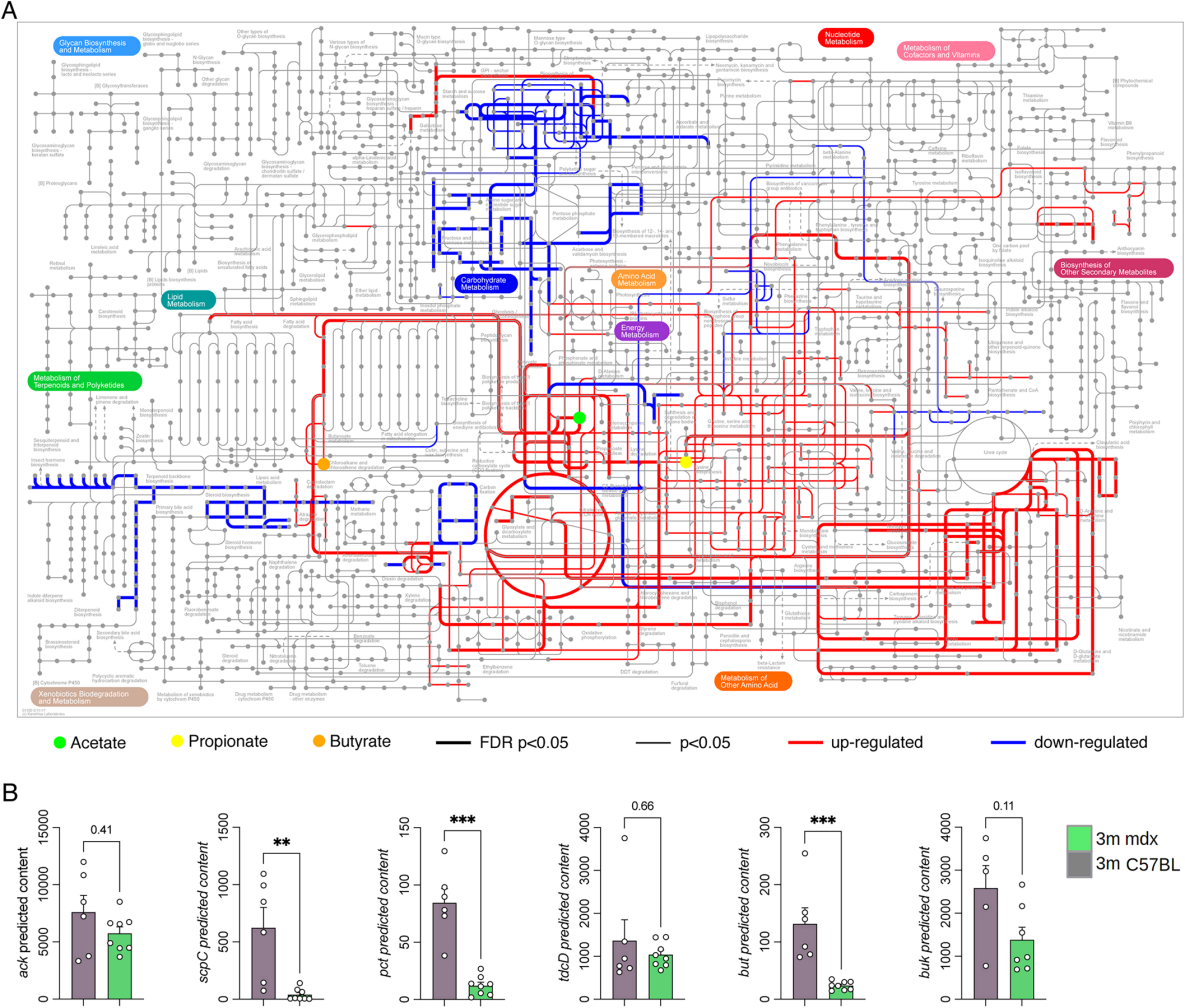
Metabolic maps of dystrophic mdx mice. (**A**) The iPath3.0 representation of KEGG metabolic pathways inferred from Piphillin analysis significantly upregulated (in red) or downregulated (in blue) in 3 m C57Bl (*n* = 6) vs 3 m mdx (*n* = 8) mice. Nodes in the map colored in green, yellow, and orange correspond to acetate, propionate, and butyrate, respectively. Line thickness represents the level of statistical significance for the inferred pathways; thick lines with FDR-corrected *P* value < 0.05, thin lines with nominal *P* value < 0.05. (**B**) Predicted metagenomic gene content of the key enzymes catalyzing the final steps for the production of microbiota-derived SCFAs. *P < 0.05, **P < 0.01, ***P < 0.001; Kruskal–Wallis test. Source data are available online for this figure.

Multidimensional scaling analysis (MDS) showed significantly different metabolic profiles among groups (Fig. 2G). Accordingly, the predicted functional potential of the mdx-associated microbiota showed alterations of metabolic pathways related to carbohydrate and amino acid metabolism (Metabolic Maps C57Bl and 3 m mdx; Dataset EV1 and Fig. 3A). Indeed, 3 m mdx mice showed a significant reduction in the predicted gene content of the key SCFA biosynthetic enzymes propionyl-CoA:succinate CoA transferase (*scpC*), propionate CoA-transferase (*pct*), butyryl-CoA:acetate CoA-transferase (*but*) and butyrate kinase (*buk*) while no differences where observed for propionate (*tdcD*) and acetate (*ack*) kinases (Fig. 3B). We observed a significant reduction in the concentration of different amino acids, namely alanine, aspartic acid, methionine, and phenylalanine, in 3 m mdx mice compared to C57Bl animals (Fig. 2H). Of note, tartarate may act as a muscle toxin by inhibiting the production of malic acid (Zhang et al, 2021).

**Figure EV1:**
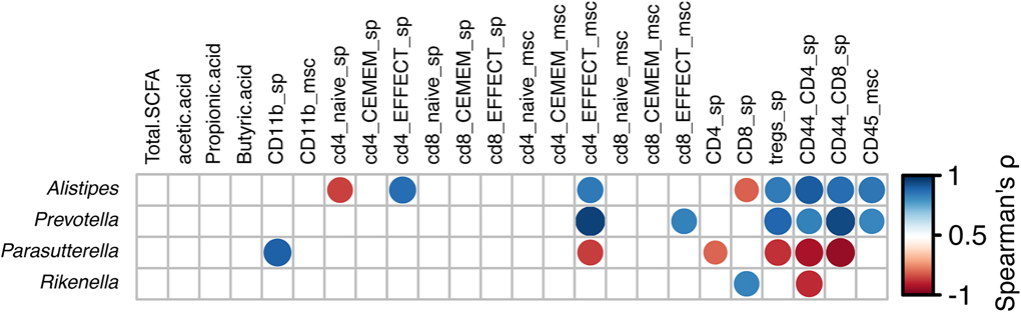
Correlation between bacterial genera and immunity. Heatmap of Spearman’s rho correlations between the relative abundance of the most represented bacterial genera (with relative abundance > 0.1%) in the gut microbiota of 3 m mdx animals (*n* = 3–8) with the indicated metabolites and immunological parameters. The significant correlations with FDR-corrected *P* value < 0.1 are indicated with bubbles. Spearman correlation plots for the significant correlations between Prevotella and the indicated immunological parameters are also shown. sp spleen-derived, msc muscle-derived, CEMEM central memory cells, EFFECT effector cells. Source data are available online for this figure.

To evaluate the extent of gut microbiota effects on systemic and muscle immunity, as well as inflammation, we correlated metataxonomic and immunophenotyping data (Dataset EV2; Fig. EV1). Specifically, we investigated the correlations among FACS analysis of different subsets of T cells (naive, central memory, effector T cells, and Tregs) and CD11b+ myeloid subset of spleen and muscle tissues in 3 m mdx and C57Bl mice and the most representative microbiota genera (Dataset EV2; Fig. EV1). We observed that *Prevotella* significantly correlated with the frequency of splenic CD44 + CD4 + /CD8 + T cells and Tregs, as well as with muscle effector/memory CD44 + CD8 + T cells and central memory CD4 + T cells (Fig. EV1).

### Gut microbiota depletion reduces innate immune response but alters muscle metabolism and function in 3-month-old mdx mice

**Figure 4:**
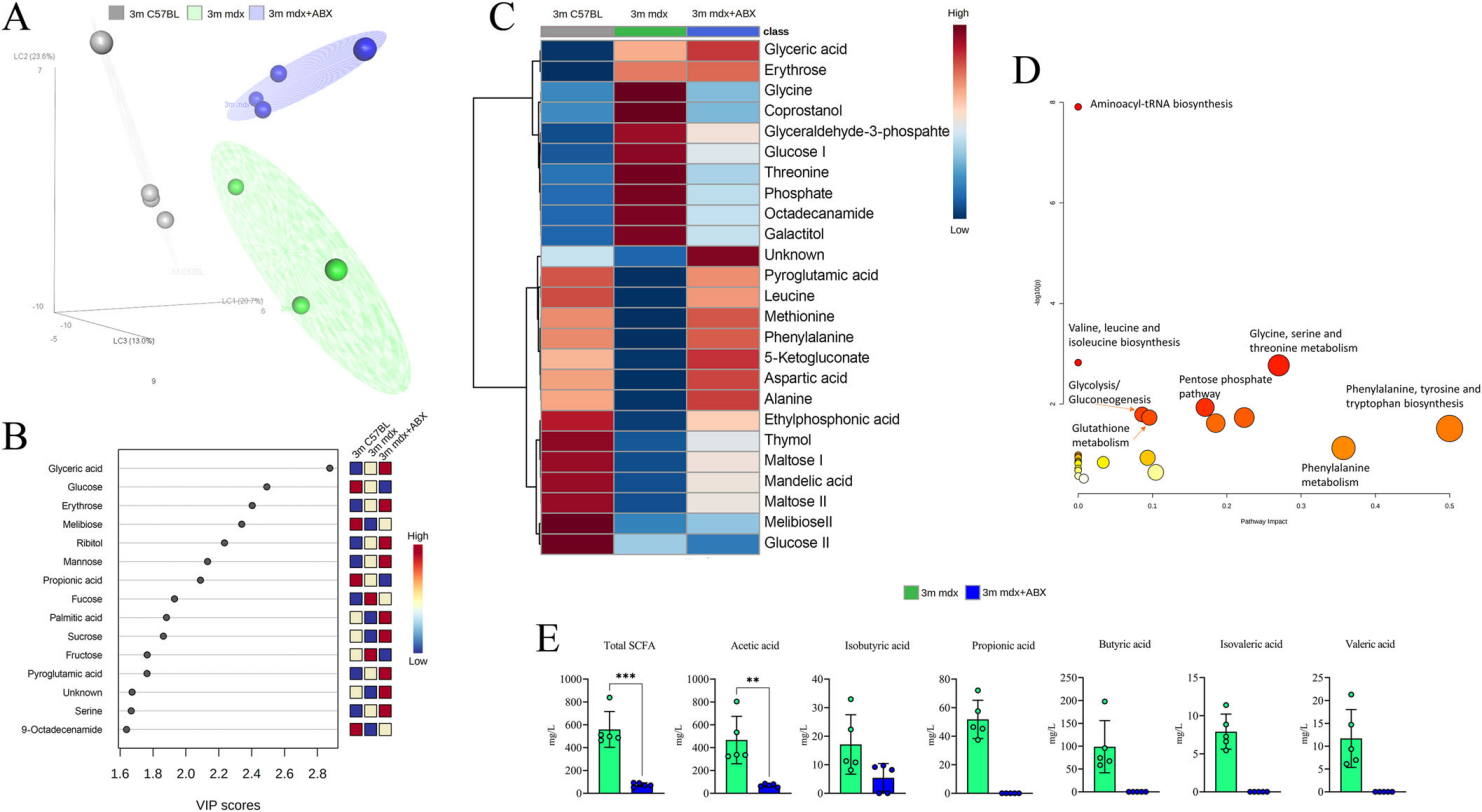
Characterization of gut tissue metabolome in 3 m mdx mice and following antibiotics treatment. (**A**) Partial least square discriminant analysis (PLS-DA) models score plot used to evaluate the differences among 3 m C57B1 (in gray), 3 m mdx mice (in green), and 3 m mdx+ABX) (dark purple), with *n *= 4 each. (**B**) Relevant metabolites (top variable importance in projection score) in the corresponding PLS-DA separation, in blue metabolites with a negative fold change and in red metabolites with a positive fold change. (**C**) Heatmap showing all the relevant metabolites concentration change among the groups. Both metabolites and classes were clusterized according to the Wald method. (**D**) Metabolic pathways involving the relevant metabolites obtained using the MetPa algorithm. The color and size of each circle are based on the *P* value and pathway impact value, respectively. The *x* axis represents the pathway impact, and the *y* axis represents the −log of *P* values from the pathway enrichment analysis for the key differential metabolites of 3 m mdx and 3 m mdx+ABX mice. (**E**) Fecal content quantification of SCFAs in 3 m mdx and 3 m mdx+ABX mice (*n* = 5 per group). Data equal to 0. Data are presented as mean ± SD (**P < 0.01, ***P < 0.001; Student *t* test). Source data are available online for this figure.

**Figure 5:**
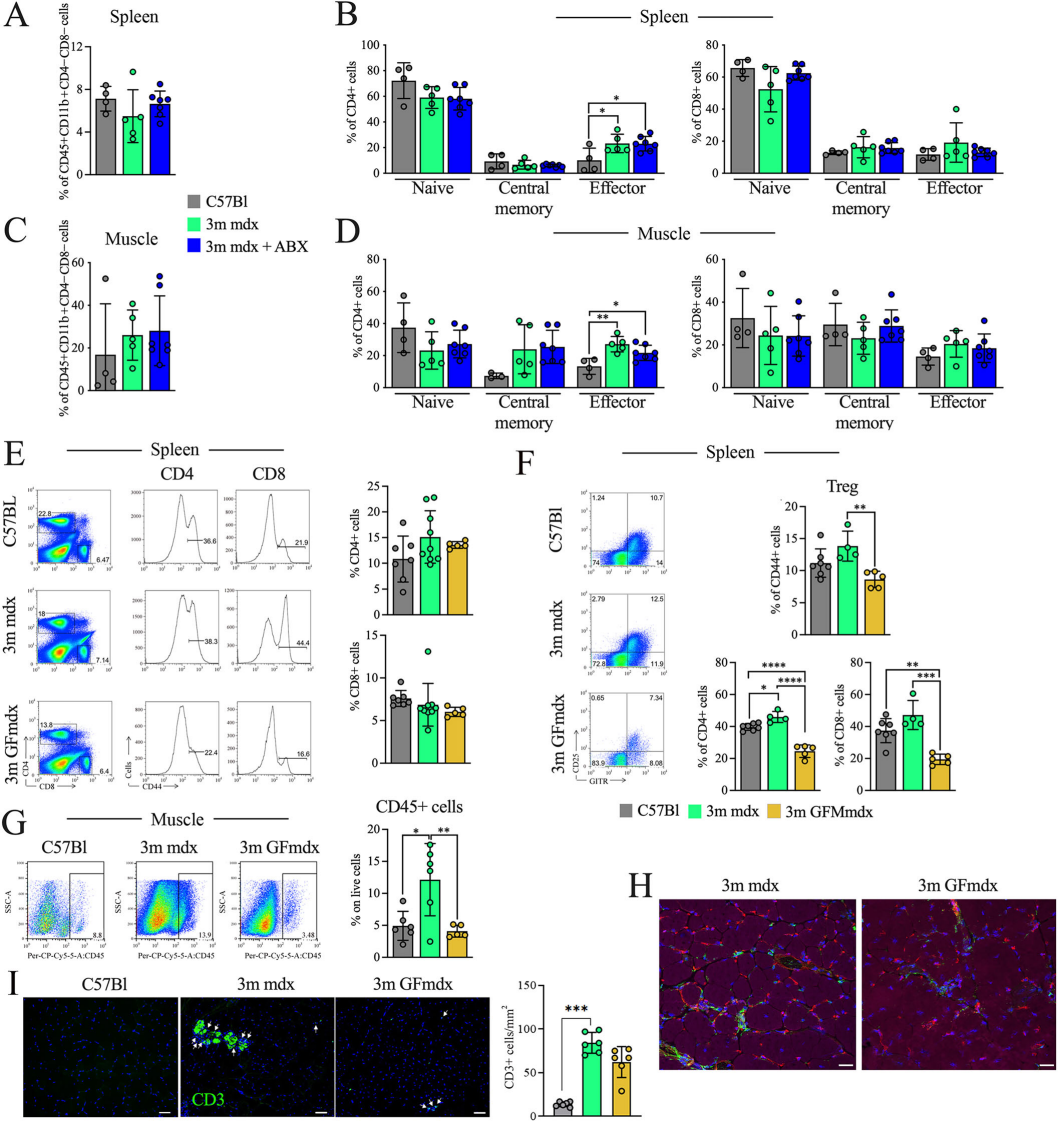
Microbiota depletion induces modulation of immune cells. (**A**–**D**) FACS analysis of spleen and muscle homogenates from 3 m C57Bl (*n* = 4), mdx (*n* = 5), and mdx+ABX (*n* = 7) mice demonstrates no significant alteration of CD45 + CD11b + CD4-CD8- myeloid cells (**A**, **C**) and few differences in CD4+ or CD8+ naive (CD62L + CD44-), central memory (CD62L + CD44 + ), and effector (CD62L− CD44 + ) T cells (**B**, **D**). (**E**) FACS analysis of spleen of 3 m C57Bl (*n* = 7), mdx (*n *= 9), and GFmdx (*n* = 5) mice revealed similar proportions of CD4+ and CD8 + T cells but reduced activated CD44 + T cells in GFmdx mice. Representative plots are depicted. (**F**) Graphs show cumulative frequencies of CD4+ and CD8 + T cells on live cells of 3 m C57Bl (*n* = 7), mdx (*n* = 4), and GFmdx (*n* = 5) mice. Representative dot plots and cumulative frequencies of splenic CD4 + GITR + CD25+ Treg. Frequencies of effector CD44 + T cells were significantly decreased in the spleen of GFmdx mice. (**G**) Representative dot plots showing the proportion of muscle-infiltrating CD45+ cells of 3 m C57Bl (*n* = 6), mdx (*n* = 6), and GFmdx (*n* = 5) mice. Cumulative frequencies of muscle-infiltrating CD45+ cells are shown. (**H**) Representative images of TA muscles from 3 m mdx and GFmdx mice stained for CD45 (in green), isolectin (in red), and phalloidin (in purple). Nuclei were counterstained with DAPI (in blue). Scale bar: 10 μm. (**I**) Absolute number of CD3+ inflammatory cells (white arrows) was quantified in *n* = 12 images of TA of 3 m C57Bl, 3 m mdx, and 3 m GFmdx mice (*n* = 6 each). CD3 staining is shown in green and DAPI in blue. Scale bars: 50 μm. Data are presented as mean ± SD. *P < 0.05; **P < 0.01; ****P < 0.0001, ordinary one-way ANOVA, Tukey’s multiple comparison test. The comparisons among the averages of CD3+ cells were evaluated using unpaired *t* test. Source data are available online for this figure.

**Figure EV2:**
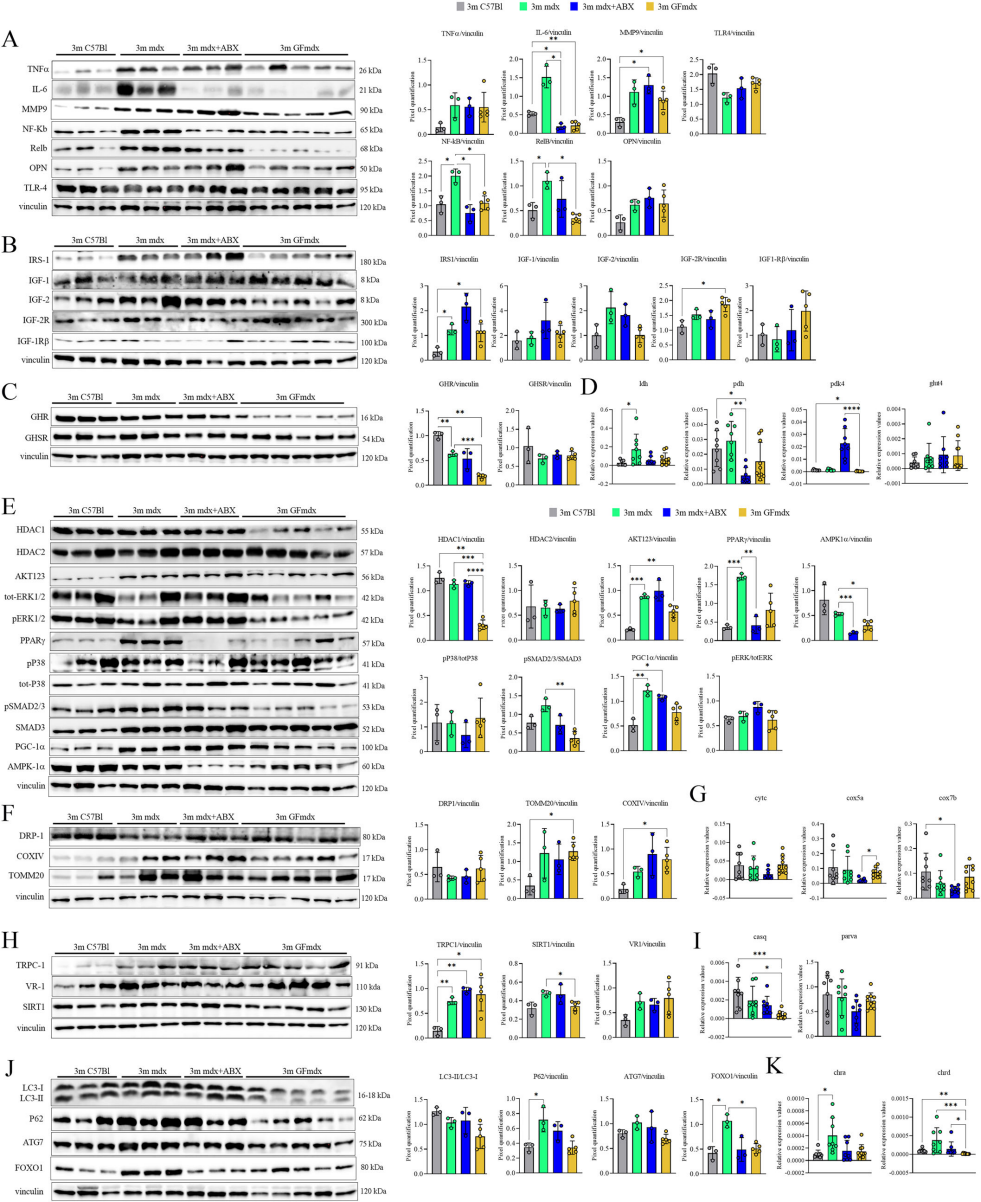
Gene and protein expression in muscles from 3 m mdx, mdx + ABX, and GFmdx. Cropped images of representative WB and RT-qPCR analysis of TA muscle of 3 m mdx (*n* = 3/4), 3 m mdx+ABX (*n* = 3/4) and 3 m GFmdx (*n* = 5) showing the expression of the proteins specifically involved in inflammation/fibrosis (**A**), skeletal muscle metabolism (**B**–**E**), mitochondrial biogenesis (**F**, **G**), calcium conducting channels (**H**, **I**), autophagy (**J**) and Nicotinic acetylcholine receptors (**K**). Densitometric data were normalized on vinculin and expressed as mean ± SD. Data are presented as mean ± SD (*P < 0.05, **P < 0.01, ***P < 0.001; ****P < 0.0001, ordinary one-way ANOVA, Tukey multiple comparison test for WB and nonparametric test followed by Kruskal–Wallis test for RT-qPCR). Source data are available online for this figure.

**Figure 6:**
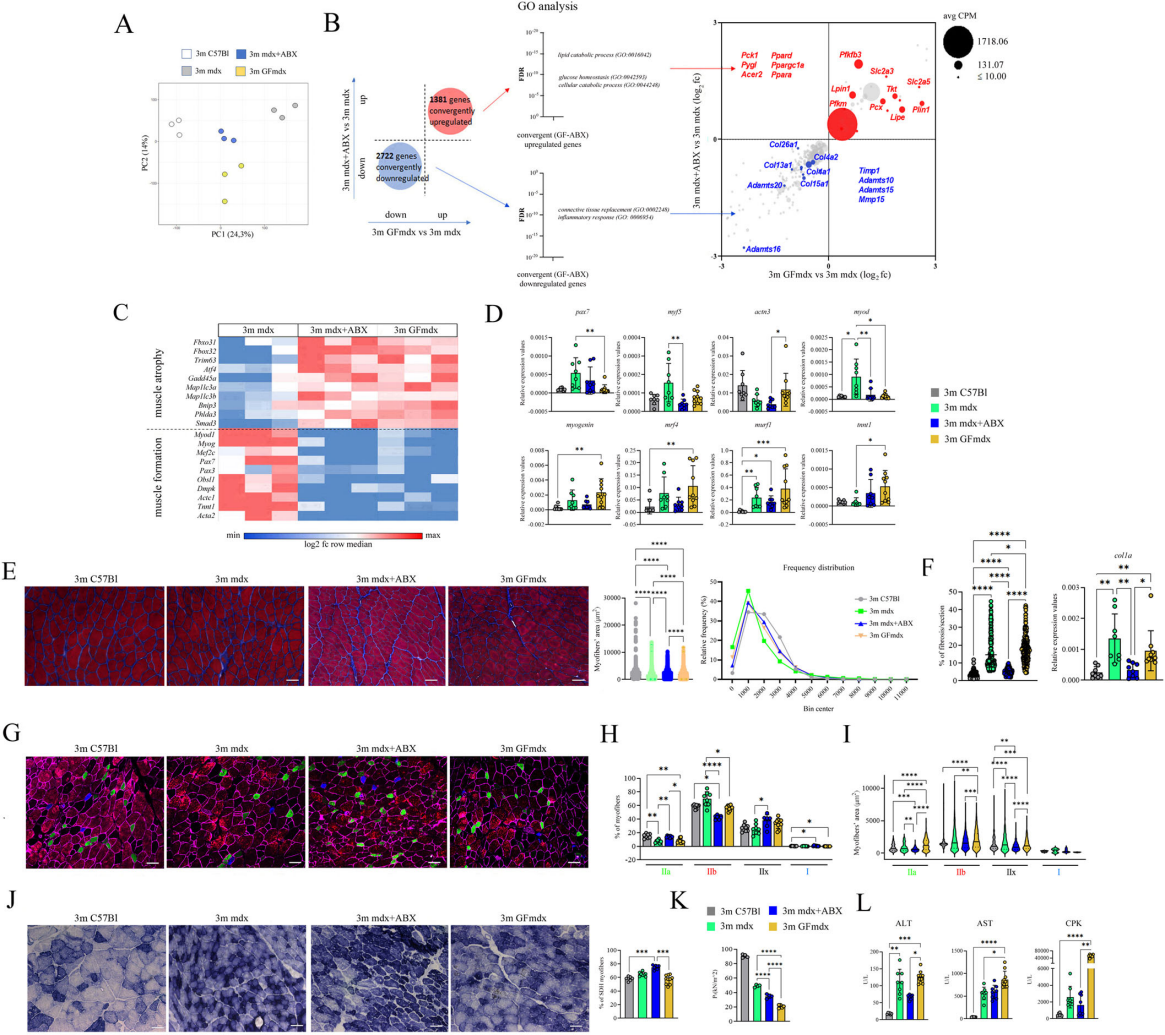
Muscle homeostasis of 3 m mdx mice is influenced by microbiota. (**A**) RNA datasets clustering and (**B**) convergently up- and downregulated genes of muscles of 3 m mdx (*n* = 3), mdx+ABX (*n* = 3), and GFmdx (*n* = 3) mice. (**C**) Gene ontology (GO) analysis on both groups of convergent genes. (**D**) RT-qPCR analysis of TA muscles of two independent experiments with 3 m mdx (*n* = 4), mdx+ABX (*n* = 4), and GFmdx (*n* = 5) mice determined the expression of myogenic markers. (**E**) Representative Gomori modified staining and quantification of myofiber area and relative frequency of the myofiber cross-sectional area (CSA) expressed as the frequency distribution of the TA muscles of 3 m C57Bl (*n* = 4), 3 m mdx (*n* = 4), mdx+ABX (*n* = 4), and GFmdx mice (*n* = 5). Pooled samples for each group with *n* = 6240 for 3 m C57Bl; *n* = 6001 for 3 m mdx; *n* = 10,556 for 3 m mdx+ABX; *n* = 23,059 for GFmdx. For morphometric analysis, images were quantified with ImageJ software for each mouse. Scale bars: 50 μm. (**F**) Quantification of the fibrotic area from Gomori-stained images (pooled samples for each group with *n* = 223 for 3 m C57Bl, 3 m mdx, and 3 m mdx+ABX; *n* = 215 for GFmdx) and RT-qPCR analysis of *Col1a* (two independent experiments with *n* = 4 animals each group). (**G**) Representative images of skeletal muscle showed the distribution and composition of the myosin heavy chain (MyHC) isoforms (Type IIa, Type IIx, and Type IIb). (**H**) Graph portrays the percentage of myofibers expressing different MyHC isoforms, and (**I**) myofibers area per type of MyHC in TAs of 3 m mdx (*n* = 4), mdx+ABX (*n* = 4), and GFmdx (*n* = 5) mice (*n* = 12 images per animal). Scale bars: 50 μm. (**J**) Representative SDH staining and quantification of percentage of SDH+ myofibers of TAs from 3 m mdx (*n* = 4), mdx+ABX (*n* = 4), and GFmdx mice (*n* = 5) (*n* = 12 images per animal). Scale bars: 50 μm. (**K**) Tetanic force of TA muscle of 3 m C57Bl (*n* = 4), mdx (*n* = 4), mdx+ABX (*n* = 4), and GFmdx (*n* = 5) mice. (**L**) ALT, AST, and CPK serum levels were measured in 3 m mdx (*n* = 4), mdx+ABX (*n* = 4), and GFmdx mice (*n* = 5) (two independent experiments). Data are presented as mean ± SD (*P < 0.05, **P < 0.01, ***P < 0.001; ****P < 0.0001, ordinary one-way ANOVA, Tukey multiple comparison test for WB and nonparametric test followed by Kruskal–Wallis test for RT-qPCR). Source data are available online for this figure.

**Figure EV3:**
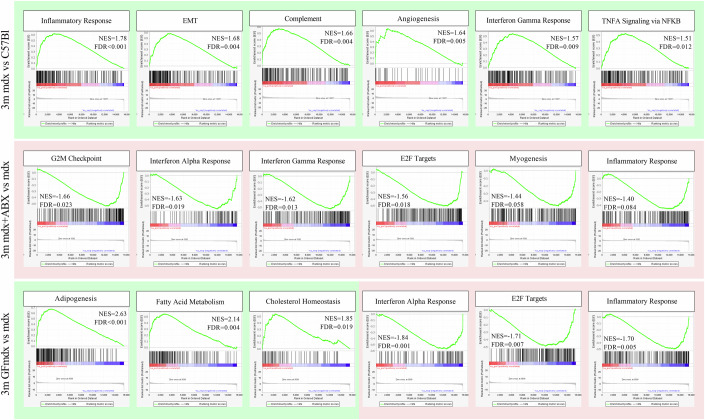
Gene set enrichment analysis (GSEA) of 3 m C57bl, mdx, mdx + ABX, and GFmdx mice RNA sequencing data. The annotated dataset “Hallmark” collection by the Molecular Signatures Database (MSigDB) was used as a reference. A green background refers to positive Normalized Enrichment Score (NES) (enrichment in positive phenotype, or upregulation); a red background refers to negative NES (enrichment in negative phenotype, or downregulation). FDR: False discovery rate. Genes involved in inflammatory response, epithelial-to-mesenchymal transition (EMT), complement activity, angiogenesis, and interferon-γ response are upregulated in 3 m mdx (*n* = 3) vs age-matched C57Bl (*n* = 3) mice (top lane). Genes involved in G2M checkpoint transition, interferon-α and -γ response, E2F transcriptional activity, and myogenesis are downregulated in 3 m mdx+ABX (*n* = 3) vs age-matched mdx (*n* = 3) mice (mid lane). Genes involved in adipogenesis, fatty acid metabolism, and cholesterol homeostasis are upregulated in 3 m GFmdx (*n* = 3) vs age-matched mdx (*n* = 3) mice; conversely, genes involved in interferon alpha response, E2F transcriptional activity, and inflammatory response are downregulated in the 3 m GFmdx vs age-matched mdx mice (bottom lane). Source data are available online for this figure.

Since mdx mice harbor alterations in the intestinal microbiome, we investigate whether early secondary effects of muscular dystrophy could be affected by the absence of gut microbiota in the GFmdx dystrophic model or by long-term depletion of intestinal bacteria of mdx by oral treatment with a cocktail of ampicillin, metronidazole, and vancomycin (ABX) (Khan et al, 2019; Tan et al, 2022). ABX can affect luminal secondary metabolites and gut signaling (Zarrinpar et al, 2018). Metabolomic analysis of the small intestine in 3 m mdx+ABX demonstrated significant alterations of the metabolic profiles and inferred pathways (FRD-corrected *P* < 0.05, ANOVA), suggesting an important role of the gut microbiota in affecting host metabolome (Fig. 4A,B). Given that the combined output of host-microbes interactions influencing host energy metabolism, development, and function of the immune system (Zheng et al, 2007) is determined by the metabolome, we performed a metabolomic analysis of the small intestinal content from mdx and mdx+ABX mice. Metabolic pathways reconstruction based on the metabolites with the highest discriminatory power (Dataset EV1; Fig. 4C,D) revealed that mdx overexpressed glycine, coprostanol, threonine, phosphate, and galacticol related to age-matched C57Bl, while leucine, methionine, aspartic acid, and alanine were downregulated. Interestingly, 3 m mdx+ABX mice showed metabolites’ expression similar to wild-type mice, except for maltose and mandelic acid, whose amount was lower in dystrophic mice related to 3 m C57Bl (Fig. 4C,D). Hence, ABX changed the SCFA pool of mdx, most notably by decreasing butyric, propionic, isopropionic, and valeric acids to undetectable levels, while acetic acid was significantly decreased when compared to untreated mdx mice (Fig. 4E). We further highlighted the influence of gut microbiota depletion on the immune response of 3 m mdx. The amount of splenic CD45 + CD11b + CD4-CD8- myeloid cells was not modified by ABX treatment (Fig. 5A) neither the effector CD4 + T cells which remained upregulated in both mdx and mdx+ABX compared to C57Bl (Fig. 5B). In muscle, we did not find differences in the CD45+ myeloid cells (Fig. 5C) since ABX only slightly modified the effector CD4 + T cells in mdx compared to untreated age-matched C57Bl (Fig. 5D). According to these evidences and to literature describing modulation of immune system and microbiota in GF mice (Round and Mazmanian, 2009), we investigated the consequences of gut microbiota depletion on the amounts of lymphocytes in 3 m GFmdx. We observed a similar amount of CD4+ and CD8 + T cells between 3 m mdx and 3 m GFmdx (Fig. 5E), whereas a significant reduction of splenic Treg cells and effector/memory CD44 + T cells (Fig. 5F) was shown in 3 m GFmdx compared to age-matched mdx mice. The amount of inflammatory CD45+ cells was also diminished in the skeletal muscle of 3 m GFmdx related to 3 m mdx mice (Fig. 5G), as well as the number of CD3+ inflammatory cells, as demonstrated by quantification of immunofluorescence staining (Fig. 5H). Compared with 3 m mdx, ABX-treated mdx and GFmdx muscles showed a significant decrease in IL-6 but not in TNFα cytokine, as well as a significant reduction of Nuclear Factor kappa-light-chain-enhancer of activated B cells (NF-kB) and RelB inflammatory mediators toward the levels of WT (Fig. EV2A), suggesting a reduced muscle innate immune response (Ticinesi et al, 2017). There was no change in the expression of TLR4 and osteopontin (OPN), but mdx, mdx+ABX, and GFmdx muscles showed an increase in matrix metallopeptidase (MMP) 9 relative to WT (Fig. EV2A). To evaluate the influence of the gut microbiota on the dystrophic skeletal muscle architecture and function, we performed RNAseq of tibialis anterior (TA) muscles from mdx, ABX-treated mdx, and GFmdx mice. Principal component analysis (PCA) of RNAseq datasets showed treatment-dependent clustering of samples, separating mdx+ABX and GFmdx from mdx (Dataset EV3; Fig. 6A). As additional quality control, mdx RNA datasets clustered separately from the ones of age-matched, background-matched C57Bl muscles (Fig. 6A). Comparing ABX-treated and GFmdx to age-matched mdx muscle, we found 1381 genes convergently upregulated and 2722 genes convergently downregulated (Fig. 6B). Gene abundance cutoff was set at 10CPM for these initial analyses to focus our comparisons on genes of mid-to-high expression in muscle. We performed gene ontology (GO) analysis on both groups of convergent genes. GO analysis of the convergent upregulated genes showed enrichment for pathways of oxidative metabolism (*Lpin1, Ppard, Ppargc1a, Ppara*) and nutrient uptake/processing (*Pfkm, Pck1, Pfkfb3, Pcx, Slc2a3, Slc2a5, Tkt, Pygl, Plin1, Lipe, Acer2*) (Dataset EV4; Fig. 6B,C). Conversely, ABX and GF treatments converged on downregulating genes involved in inflammation and fibrosis (*Timp1, Mmp15*, several members of the *Adamts* extracellular proteases, and *Collagen* gene families) (Dataset EV4; Fig. 6B,C). Furthermore, gene set enrichment analysis (GSEA) revealed alterations in inflammatory response, epithelial-to-mesenchymal transition (EMT), complement activity, angiogenesis, and interferon-γ response in 3 m mdx vs age-matched C57Bl muscles (Fig. EV3). Moreover, genes involved in G2M checkpoint transition, interferon-α and -γ response, E2F transcriptional activity, and myogenesis were reduced in 3 m mdx+ABX versus age-matched mdx muscles (Fig. EV3). Muscle genes involved in adipogenesis, fatty acid metabolism, and cholesterol homeostasis were upregulated in 3 m GFmdx vs age-matched mdx mice; conversely, muscle genes involved in interferon-αresponse, E2F transcriptional activity, and inflammatory response were downregulated in the 3 m GFmdx vs age-matched mdx mice (Fig. EV3).

We further validated muscle RNAseq analysis for genes involved in myogenesis. Compared with mdx, mdx+ABX and GFmdx muscles showed decreases in genes involved in early myogenesis as *MyoD*, *Pax7*, and *Myf5* with similar levels of later genes of myogenesis as *myogenin* and *MRF4* (Fig. 6D). Relative to WT controls, mdx+ABX and GFmdx muscles showed no difference in expression for the majority of these genes except for *myogenin* and *MRF4*, where GFmdx muscles showed significant increases (Fig. 6D). Transcript expression of the E3 ubiquitin ligase gene, *MuRF-1*, which promotes protein degradation and muscle catabolism (Lahiri et al, 2019), was similarly and significantly increased in mdx, mdx+ABX and GFmdx muscles compared with WT (Fig. 6D). These results suggest that microbiota depletion may likewise alter the regulation of genes related to muscle growth and differentiation in mdx.

To uncover the effects of changes seen in microbiota-depleted dystrophin-deficient mice, we examined the muscle cross-sectional areas (CSAs) of mdx+ABX and GFmdx compared to age-matched mdx and WT mice. The ABX-treated mdx and GFmdx displayed increased CSAs relative to mdx, with the former presenting the highest area of myofibers (mean fiber area for TA ± SEM: 3 m mdx 1625.01 ± 19.59 μm^2^; 3 m mdx+ABX 1855.572 ± 11.903 μm^2^; 3 m GFmdx: 1754.22 ± 7.937 μm^2^; 3 m C57Bl 1998.21 ± 16.79 μm^2^), and the latter a lower size variability (Fig. 6E). In particular, the values of frequency distribution confirmed the smaller area of myofibers in 3 m mdx mice (25% Percentile: 3 m mdx: 661.68; 3 m mdx+ABX: 1003.75; 3 m GFmdx: 838.447; 3 m C57Bl: 1131.217. 75% Percentile: 3 m mdx: 2061.384; 3 m mdx+ABX: 2443.35; 3 m GFmdx: 2398.803; 3 m C57Bl: 2621.3125) (Fig. 6E). Reduced immune response correlated with reduced fibrosis deposition in mdx+ABX and GFmdx (% of fibrosis per muscular section: 3 m mdx 20.62%; 3 m mdx+ABX 5.016%; 3 m GFmdx 17.58%) (Fig. 6F). As an additional confirmation of the RNAseq data, RT-qPCR analysis for fibrotic genes confirmed the downregulation of *col1a* in mdx+ABX compared to age-matched mdx and GFmdx (Fig. 6F).

To determine whether these features were associated with fiber switch, the immunoreactivity for adult myosin heavy chain isoforms (MyHC) was detected and quantified by immunofluorescence (Fig. 6G). Compared to mdx, the percentage of oxidative MyHC-I and oxidative/glycolytic MyHC-IIa/IIx fibers remained unchanged in GFmdx, whereas the percentage of type IIa and IIx fibers significantly increased in mdx+ABX (Fig. 6H). Interestingly, mdx+ABX and GFmdx showed a significant increase in type I fibers relative to WT and a significant reduction of glycolytic MyHC-IIb fibers compared to mdx (Fig. 6H). Consistent with these findings, there was no change in the expression of *α-actinin-3 (ACTN3)*—a sarcomeric protein mainly expressed in fast type-IIb myofibers—except for significant differences between mdx+ABX and GFmdx which correspond to their relative proportions of type IIb fibers (Fig. 6D). Moreover, the oxidative switching of myofibers in mdx+ABX and GFmdx was confirmed by a trend for increased expression of *slow skeletal muscle troponin T-1* (*TNNT1*) compared to mdx and WT (Fig. 6D). Interestingly, compared to mdx, the myofiber areas of type IIb and IIx fibers were decreased in both 3 m mdx+ABX and 3 m GFmdx mice, whereas type IIa area was increased in 3 m GFmdx and decreased in 3 m mdx+ABX (Fig. 6I). We further evaluated the metabolic activity of microbiota-depleted mdx muscle by staining for succinate dehydrogenase (SDH) activity, indicative of oxidative metabolism (Hardee et al, 2021; Schill et al, 2016). The ABX-treated mdx muscles showed a higher percentage of SDH-positive fibers, in favor of higher oxidative metabolism than 3 m GFmdx and WT (Fig. 6J). These data supported a shift in fiber type toward an oxidative phenotype in microbiota-depleted mdx, exacerbating the hallmark of muscular dystrophy, through a fiber-type switch from damage-sensitive glycolytic type IIb fibers toward damage-resistant glycolytic-oxidative type IIx and IIa fibers. In addition, ABX and GF conditions differentially affect change toward a slower MyHC profile: unlike germ-free conditions, under which microbiota depletion is maintained throughout life, allowing gradual plastic remodeling in response to metabolic changes, ABX-treated muscles might undergo a substantial increase in muscle catabolism, as suggested by the reduced size of the more abundant glycolytic-oxidative fibers.

The decrease in tetanic force is a hallmark of muscular dystrophies. We noticed a significant reduction of tetanic force in the TA muscle of both mdx+ABX and GFmdx compared to mdx and WT. However, the TA tetanic force of GFmdx was significantly lower than the force exerted by mdx+ABX (Fig. 6K). To better understand muscular weakness observed in ABX-treated mdx and GFmdx mice, we investigated serum concentration of alanine aminotransferase (ALT) and aspartate aminotransferase (AST): both ALT and AST were upregulated in GFmdx (Fig. 6L). Of note, we found an increase of creatine phosphokinase (CPK) in 3 m GFmdx versus mdx+ABX and WT, suggesting an important damage of skeletal muscle membranes (Fig. 6L).

Muscle metabolism modifications induced by depletion of mdx microbiota prompted us to analyze the insulin-like signaling and the orexigenic gut-peptide hormone ghrelin (GHR), which is known to affect whole-body energy metabolism. Among insulin-like growth factor (IGF) pathways, we detected a significant upregulation of the insulin receptor substrates 1 (IRS-1), a key modulator of insulin resistance (Gual et al, 2005), in muscles of GFmdx and mdx relative to wild-type mice (Fig. EV2B). Moreover, we showed that ghrelin was downregulated in muscles of GFmdx compared to mdx and WT (Fig. EV2C). We thus measured the expression of the mitochondrial pyruvate dehydrogenase lipoamide kinase isozyme 4 (*pdk4*), whose activity is regulated by insulin and is necessary to decrease glycolytic metabolism and conserve glucose (Pettersen et al, 2019). The *pdk4* was upregulated in mdx+ABX related to mdx and GFmdx, whereas no differences were found in the expression of glucose transporter *glut4* (Fig. EV2D), which is involved in the uptake of lactic acid in oxidative fibers for oxidation (Kellogg et al, 2017). Consistent with this, we observed a trend for decreased expression of lactate dehydrogenase (*Ldh*) and pyruvate dehydrogenase (*Pdh*) in mdx+ABX and GFmdx compared to mdx (Fig. EV2D). Notably, inhibition of Pdh activity by Pdk4 reduces the conversion of glycolytically derived pyruvate into acetyl-CoA, thereby diverting glucose flux to lactate and away from oxidation in the TCA cycle (Jeoung and Harris, 2010). All these data suggest that microbiota depletion in mdx induces alterations in cellular glucose metabolism recognized as aerobic glycolysis (Locasale and Cantley, 2011).

To further unravel a potential role of microbiota depletion in altering muscle glucose uptake and fatty acid oxidation of mdx, we investigated the downstream signaling of AMP-activated protein kinase (AMPK). Histone deacetylase (HDAC) activity is partly modulated through activation of AMPK (Vancura et al, 2018). Downregulation of HDAC1 and similar amounts of HDAC2 were observed in GFmdx mice (Fig. EV2E). Compared with mdx, both ABX-treated mdx and GFmdx mice exhibited reduced muscle 5’-AMP-activated protein kinase catalytic subunit alpha-1 (AMPK-1α) and downregulation of the peroxisome proliferator-activated receptor gamma (PPARγ) and Small mother against decapentaplegic 2/3 (SMAD2/3) that acts as a fatty acid sensor to control adipogenesis (Fig. EV2E). The reduction of AMPK-1α observed in muscles of mdx+ABX and GFmdx was associated to unmodified levels of the insulin-dependent downstream pathways that control energy homeostasis, including serine–threonine protein kinase 1-2-3 isoform (AKT 1-2-3) and extracellular signal-regulated kinase (ERK) (Fig. EV2E). Among downstream targets of AMPK, we found a significant increase of peroxisome proliferator-activated receptor gamma coactivator 1α (PGC1α) without modifications of p38 mitogen-activated protein kinases (p38 MAPKs) in mdx, mdx+ABX, and GFmdx relative to WT muscles (Fig. EV2E). In accordance with the increase of PGC1α, which is a master regulator of mitochondrial biogenesis and function (Fernandez-Marcos and Auwerx, 2011), we found a trend for increased mitochondrial mass revealed by translocase of outer mitochondrial membrane 20 (TOMM-20) and for increased mitochondrial activity identified by cytochrome c oxidase (COX) IV in mdx, mdx+ABX, and GFmdx relative to WT muscles (Fig. EV2F). GTPase dynamin-related protein 1 (DRP1), which is critical for mitochondrial fission machinery and mitochondrial dynamics, was not affected (Fig. EV2F). However, ABX-treated mdx muscle displayed downregulation of mitochondrial genes, such as *CoxVa*, *CoxVIIb*, but not *cytc*, related to 3 m GFmdx (Fig. EV2G).

Since muscle calcium dysfunctions are common in DMD (Burr and Molkentin, 2015; Mareedu et al, 2021; Matsumura et al, 2011), we investigated the amount of calcium channel proteins in muscle, such as transient receptor potential canonical 1 (TRPC1) and vanilloid receptor 1 (VR-1). Compared with WT, the TRPC1 expression was similarly upregulated in mdx, mdx+ABX, and GFmdx, whereas VR-1 was comparable among animal groups. Interestingly, sirtuin 1 (SIRT1) was found to be downregulated in GFmdx vs mdx (Fig. EV2H). We further evaluated the expression of calcium ion-binding genes, such as parvalbumin (*Pvalb*) and calsequestrin 1 (*Casq1*), whose activities mediate calcium contraction and release in the lumen of sarcoplasmic reticulum (SR) of muscle fibers: only *Casq1* was significantly downregulated in 3 m GFmdx compared to mdx and WT (Fig. EV2I).

AMP-activated protein kinase activation is also involved in autophagy activation signaling (Li and Chen, 2019). In response to nutrient deficiency and exercise, AMPK increases autophagy activity by activating Forkhead box (FOX) O1 (Saline et al, 2019). The FOXO transcription factors, including FOXO1, FOXO3, and FOXO4, have recently been implicated as key regulators of gene expression during skeletal muscle atrophy (Sandri et al, 2004), and FOXO1 mRNA in particular is upregulated during fasting and dexamethasone treatment (Chen et al, 2011). Consistent with the decrease of AMPK determined by microbiota depletion, we found a trend for reduced ratio of autophagy marker light chain 3-I and II (LC3-I/LC3-II) in GFmdx with no modifications of autophagy receptor (P62) and autophagy-related 7 (ATG7) in all groups (Fig. EV2J). However, FOXO1 was diminished in both ABX-treated mdx and GFmdx compared to age-matched mdx (Fig. EV2J). As microbiota depletion was found to regulate the neuromuscular junction (NMJ), we studied AChR genes. As reported previously by others (Mazhar and Herbst, 2012), we found downregulation of fast-channel acetylcholine receptor subunits in microbiota-depleted mdx (Fig. EV2K).

All these data highlighted that AMPK-related pathways could be the mediator of microbiota depletion in exacerbating the dysmetabolic hallmarks of DMD, such as deregulation of muscle glucose uptake and enhanced fatty acid oxidation with consequent shift in fiber type toward an oxidative phenotype.

### Dysbiotic microbiota of mdx affects intestinal, spleen, and muscle inflammation and inversely correlates with muscle function

An important characteristic of the gut microbiota is its ability to modulate host immune responses (Geva-Zatorsky et al, 2017). To determine whether restoration of microbial dysbiosis of mdx normalizes inflammatory responses, ABX-treated 3 m mdx mice were colonized with eubiotic microbiota of age-matched C57Bl (ABX-mdx^FMT_C57Bl^) via fecal microbiota transplant (FMT). The lamina propria (LP) immune cell compositions from the entire colon of ABX-mdx^FMT_C57Bl^ mice were analyzed using flow cytometry.

**Figure 7:**
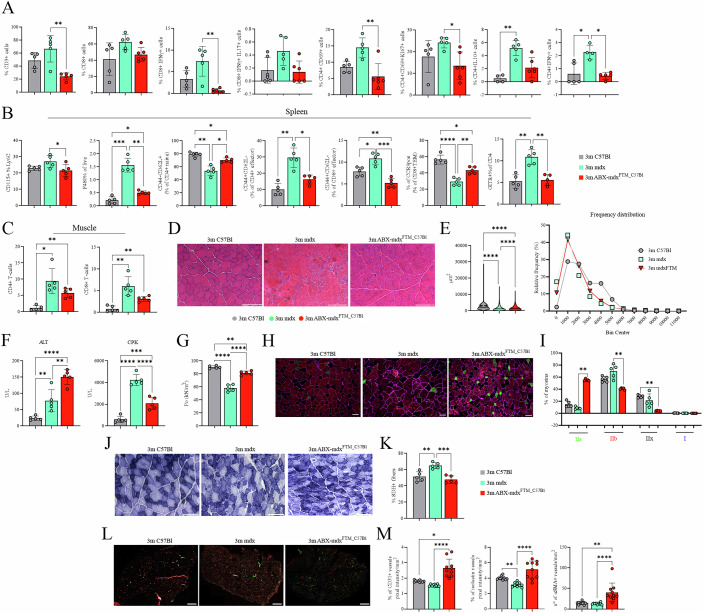
Effects of dysbiotic microbiota of mdx on intestinal, spleen, and muscle inflammation and muscle function. (**A**) FACS analysis of T cell subsets from lamina propria in 3 m C57Bl (*n* = 5), mdx (*n* = 5), and ABX-mdx^FMT_C57Bl^ (*n* = 5/6) showing a decrease in CD3 + T cells in ABX-mdx^FMT_C57Bl^. Infiltrating CD3 + CD4 + , and regulatory CD69+ subsets of CD4+ and CD8+ were decreased in ABX-mdx^FMT_C57Bl^. Eubiotic FMT in mdx modulates T helper response, with reductions in the cumulative frequencies of CD4 + IFNγ + (Th1) and CD4 + IL-10+ cells in ABX-mdx^FMT_C57Bl^. Data are presented as mean ± SD (*P < 0.05; **P < 0.01; ****P < 0.0001 ordinary one-way ANOVA, Tukey’s multiple comparison test. (**B**, **C**) FACS analysis of spleen and muscle homogenates from 3 m C57Bl (*n* = 5), mdx (*n* = 5), and ABX-mdx^FMT_C57Bl^ (*n* = 5/6). Analysis of the spleen revealed downregulation of Ly6C+ inflammatory monocytes and F4/80+ macrophages in ABX-mdx^FMT_C57Bl^. Eubiotic FMT in mdx mice determined a decrease of CD4 + /CD8 + CD44 + CD62L effector and GITR + CD4 + T cells in ABX-mdx^FMT_C57Bl^. Gut-derived CCR9 + CD8 + TEM+ cells were increased in ABX-mdx^FMT_C57Bl^. Data are presented as mean ± SD (*P < 0.05; **P < 0.01; ****P < 0.0001 ordinary one-way ANOVA, Tukey’s multiple comparison test. (**C**) Graphs showing cumulative frequencies of muscle-infiltrating CD45 + CD4+ and CD45 + CD8+ cells were decreased in ABX-mdx^FMT_C57Bl^ related to mdx mice. Representative H&E staining (**D**) and quantification of myofiber area with ImageJ software (**E**) of TA muscles from 3 m C57Bl (*n* = 5), mdx (*n* = 5), and ABX-mdx^FMT_C57Bl^ (*n* = 6). Scale bars for H&E: 200 μm. (**F**) Measurement of ALT and CPK in the serum of 3 m C57Bl (*n* = 5), mdx (*n* = 5), and ABX-mdx^FMT_C57Bl^ (*n* = 6). (**G**) Tetanic force of TA muscles from mdx (*n* = 5) and ABX-mdx^FMT_C57Bl^ (*n* = 6). (**H**) Representative images of skeletal muscle showed the distribution and composition of MyHC isoforms (Type IIa, Type IIx, and Type IIb). (**I**) The graph portrays the percentage of myofibers expressing different MyHC isoforms. *n* = 12 images were analyzed for each mouse. Scale bar: 50 μm. (**J**, **K**) Representative SDH staining and quantification of percentage of SDH+ myofibers of TA muscles from mdx (*n* = 5) and ABX-mdx^FMT_C57Bl^ (*n* = 6) (*n* = 12 images per mouse). Scale bar: 200 μm. (**L**, **M**) Representative image of CD31 (in cyan), α-SMA (in green), and isolectin (in red) staining and their quantification in TA muscles from mdx (*n* = 5) and ABX-mdx^FMT_C57Bl^ (*n* = 6) mice. Scale bar: 500 μm. Data for tetanic force, ALT, and CPK concentration and staining quantification are presented as mean ± SD (*P < 0.05, **P < 0.01, ***P < 0.001; ****P < 0.0001, one-way ANOVA, Tukey’s multiple comparison test). Source data are available online for this figure.

**Figure EV4:**
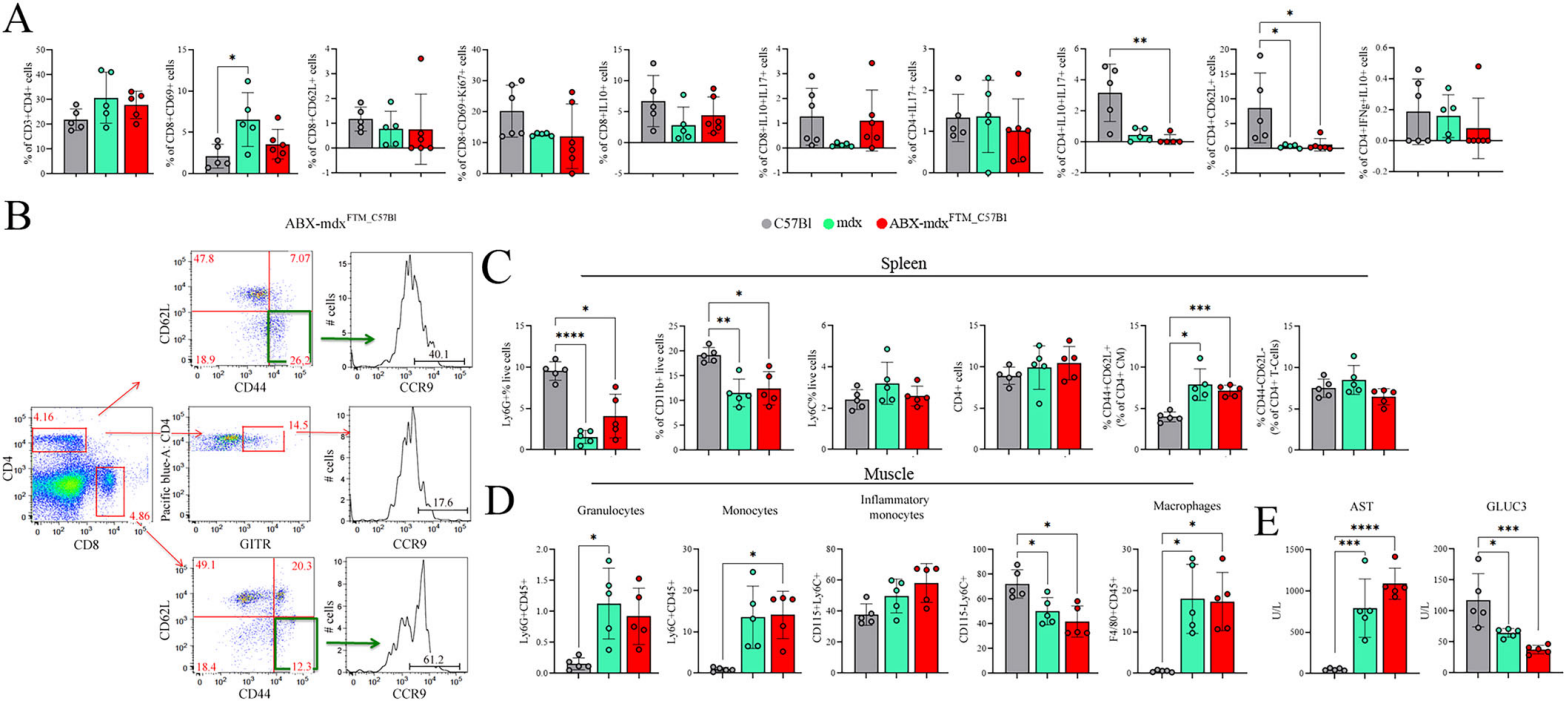
Effects of dysbiotic microbiota of mdx on intestinal, spleen, and muscle inflammation. (**A**) FACS analysis of colon lamina propria of mdx (*n* = 5) and ABX-mdx^FMT_C57Bl^ (*n* = 5/6) for quantification of T-cell subsets. (**B**) Representative plots of FACS analysis for the expression of CCR9 in ABX-mdx^FMT_C57Bl^ and ABX-C57Bl^FMT_mdx^ are depicted. The numbers within the panels indicate the percentage of each population of live cells. Each analysis included at least 5–10 × 10^4^ events for each gate. FACS analysis of T cells of spleen (**C**) and granulocyte, monocyte, and macrophage of muscle (**D**) tissues from mdx (*n* = 5) and ABX-mdx^FMT_C57Bl^ (*n* = 5/6). (**E**) Serum levels of AST and GLUC3. Data are presented as mean ± SD (*P < 0.05; **P < 0.01; ****P < 0.0001, ordinary one-way ANOVA, Tukey’s multiple comparison test). Source data are available online for this figure.

Colon LP T-cell repertoire characterization revealed a significant decrease of CD3 + T cells in ABX-mdx^FMT_C57Bl^ (Fig. 7A). Specifically, colon LP CD8 + T cells, but not CD4 + , were reduced in ABX-mdx^FMT_C57Bl^ (Figs. 7A and  EV4A). Likewise, reductions were seen in IFNγ (Th1) or IL-17 (Th17) production of colon LP pro-inflammatory CD8 + T cells in ABX-mdx^FMT_C57Bl^ (Fig. 7A). Otherwise, central memory CD62+ and activated CD69+ colon LP CD8 + T cells were similar between mdx and ABX-mdx^FMT_C57Bl^ (Fig. EV4A). Furthermore, we measured reduced activated (CD69 + CD4+ and CD69 + Ki67 + CD4 + ) and pro-inflammatory (IFNγ + CD4+ and IL-10 + CD4 + ) CD4 + T cells in the colon LP of ABX-mdx^FMT_C57Bl^ mice, whereas there were no differences in central memory CD4 + CD62+ and CD4 + Th17IL10+ in the colon LP between mdx and ABX-mdx^FMT_C57Bl^ mice (Fig. EV4A). Altogether, these data strongly support the correction of innate immune activation of CD4 + /CD8 + T cells in the colon LP of mdx via eubiotic FMT.

As a specialized immune organ, the spleen immune system plays a significant role in innate and adaptive immunity. Analysis of splenic tissue revealed that inflammatory F4/80+ macrophages as well as naive CD4 + T cells and effector memory CD4 + /CD8 + T cells (TEM) were significantly reduced in ABX-mdx^FMT_C57Bl^ as compared to mdx mice (Fig. 7B). In agreement with the normalization of the effector T-cell compartment CD4 + GITR+ regulatory T cells were significantly less abundant in the ABX-mdx^FMT_C57Bl^ mice than in mdx mice. The notable exception was the gut-derived subset of CCR9 + CD8 + TEM+, which was significantly increased in ABX-mdx^FMT_C57Bl^ compared to mdx mice (Figs. 7B and EV4B). Since eubiotic FMT may have profound effects on inflammatory responses of dystrophic muscle, we further characterized the muscles of ABX-mdx^FMT_C57Bl^ mice. No differences in granulocytes, monocytes, and macrophages percentages were observed (Fig. EV4D); however, muscle CD45 + CD4+ and CD45 + CD8+ cells were decreased in ABX-mdx^FMT_C57Bl^ mice compared with mdx mice (Fig. 7C), suggesting the amelioration of inflammation in dystrophic muscle tissues.

Morphometric analysis of TAs of ABX-mdx^FMT_C57Bl^ mice showed increased CSAs of the myofibers (mean fiber area ±SEM: 3 m mdx 1644.77 ± 18.86 μm^2^, 3 m C57Bl 1757.41 ± 9.87 μm^2^, ABX-mdx^FMT_C57Bl^ 1741.80 ± 8.33 μm^2^) and reduced fibrotic infiltrate suggesting an amelioration of the dystrophic phenotype (Fig. 7D). The values of frequency distribution confirmed the higher area of myofibers in ABX-mdx^FMT_C57Bl^ mice related to untreated age-matched mice (25% Percentile: 3 m mdx: 648.221; 3 m C57Bl: 911.385; 3 m ABX-mdx^FMT_C57Bl^: 837.8725; 75% Percentile: 3 m mdx: 2168.295; 3 m C57Bl: 2328.3305; 3 m ABX-mdx^FMT_C57Bl^: 2293.675) (Fig. 7E).

Furthermore, ALT serum level was higher in ABX-mdx^FMT_C57Bl^ mice than in mdx mice, suggesting increased lipid metabolism after eubiotic FMT (Fig. 7F), whereas similar levels of AST were found in both groups of mice (Fig. EV4E). Of note, CPK values were significantly reduced in ABX-mdx^FMT_C57Bl^ (Fig. 7F). Strength evaluation demonstrated a significant increase in tetanic force of the TA muscle in ABX-mdx^FMT_C57Bl^ mice compared to untreated mdx mice (Fig. 7G).

Consistent with the increase of myofibers area, we observed rescue of the number of oxidative/glycolytic MyHC type IIa myofibers in TAs of ABX-mdx^FMT_C57Bl^ to levels observed in healthy C57Bl (Fig. 7H,I). To further support this evidence, we analyzed SDH levels and observed a significant decrease in oxidative SDH+ fibers in ABX-mdx^FMT_C57Bl^ compared to mdx mice (Fig. 7J,K). These data support the hypothesis that correction of dysbiotic *Prevotella*-enriched microbiota in ABX-mdx^FMT_C57Bl^ promotes amelioration of skeletal muscle carbohydrate uptake and metabolism.

**Figure EV5:**
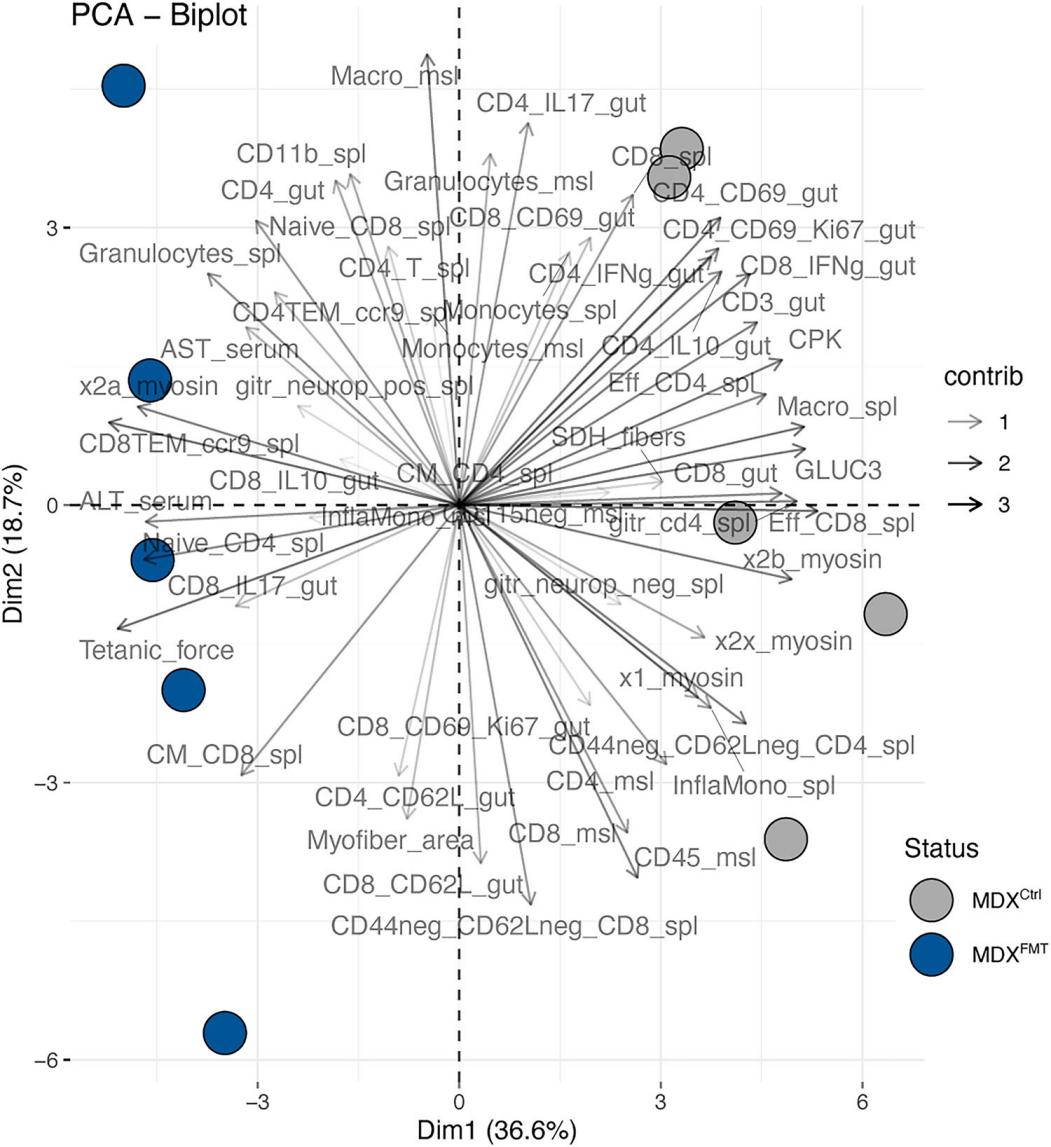
Principal component analysis (PCA) biplot of samples and analyzed variables from spleen, gut, and muscle. The biplot shows the PCA scores of the explanatory variables as vectors and samples colored according to treatment and genetic backgrounds. The color intensity of the vectors (lines) shows the strength of their contribution to each PC. Vectors pointing in similar directions indicate positively correlated variables, vectors pointing in opposite directions indicate negatively correlated variables.

By analyzing all the measured variables in combination, we observed that ABX-mdx^FMT_C57Bl^ clustered apart from mdx animals and inversely correlated with gut IFNγ-producing pro-inflammatory, proliferating, and activated CD4 + T cells as well as splenic and muscle-infiltrating T cells (Fig EV5), suggesting that eubiotic FMT modulates the immune response of mdx.

We further sought to investigate whether amelioration of sustained immunity in ABX-mdx^FMT_C57Bl^ mice muscles might be in part driven by rescue of altered vessels. Previous examination of the mdx muscle microvasculature showed a defective endothelial layer and reduced vessels (Bella et al, 2020). Accumulating evidence has shown that the gut microbiome can influence the balance of vascular homeostasis (Battson et al, 2019; Brunt et al, 2019; Kiouptsi and Reinhardt, 2018; Maiuolo et al, 2022). Staining of whole TA muscle sections of ABX-mdx^FMT_C57Bl^ mice demonstrated that eubiotic FMT efficiently increased the number of CD31+ capillaries and small arterioles expressing α-smooth muscle actin (αSMA) (Fig. 7L,M). Importantly, we found a significant increase in total isolectin-positive vessels of ABX-mdx^FMT_C57Bl^ mice (Fig. 7L,M).

All these data suggested that restoration of gut microbiota composition improves muscle pathology and function in dystrophic mdx.

## Discussion

The gut microbiota is a central player in shaping and modulating immune system responses, with gut microbial dysbiosis linked to several autoimmune and immune-mediated diseases (Kosiewicz et al, 2011). The commensal population that constitutes the microbiota is extremely variable among individuals but functionally stable (Human Microbiome Project, 2012), and its composition is also dependent on the immune responses that are mediated in the gut and on host genotypes/phenotypes (Spor et al, 2011). The interactions between the microbiota and intestinal immune cells are so strictly and hierarchically regulated that their dysfunctions cause serious problems, such as a chronic inflammatory state, contributing to alter homeostasis even at distant sites, including skeletal muscles (Kabat et al, 2016). As immune system dysregulation was recently shown as a fundamental component of DMD pathogenesis, we investigated the gut microbiota composition of dystrophic mice and its effects on muscle metabolism and function.

In DMD patients, the lack of dystrophin and dystrophin glycoprotein complex (DGC) proteins is likely to cause dysfunctions in the structural integrity of the intestinal barrier. Altered bowel permeability can represent a source of molecules that might produce muscle wasting by entering the circulation, via a disrupted gut barrier, and traveling toward muscles. In addition, this condition could be responsible for the uncontrolled passage of components of the microbiota towards the immune cells of the *lamina propria*, where they induce the pathological activation of these cells. Recent studies showed that chronic inflammation is partially dependent on the tissue-environment interface; in the mucosa of the gastrointestinal tract, antigen-presenting cells and lymphocytes drive innate and adaptive responses whose deregulation determines the rise of inflammatory events that are the common features of muscular dystrophies. Dietary metabolites function as immune-modulators: accordingly, the identification of bacteria involved in triggering the inflammation-driven muscular degeneration could open the door to manipulating these taxa for therapeutic purposes.

For the first time, we demonstrated that microbiota composition in 3 m mdx is different from age-matched C57Bl and, more importantly, we found a reduction in diversity and a selection of a few bacterial species. Of note, family-level *Prevotellaceae*, genus-level *Prevotella* were highly associated with dystrophic mdx mice. *Prevotella* has been associated with either good or bad metabolic outcomes and, probably, both depend on the presence of metabolic and inflammatory alterations (Cani, 2018). *Prevotella* was more abundant in patients suffering from untreated rheumatoid arthritis than in healthy individuals, with a reduction in the abundance of beneficial microbes. In a mouse model of gut inflammation, animals colonized with *Prevotella* had more severe disease than controls, consistent with a pro-inflammatory function of this organism (Scher et al, 2013). Interestingly, the *Prevotella*-dominated microbiome is a community shift away from *Bacteroides*, previously reported to be associated with an anti-inflammatory state and Treg production (Pandiyan et al, 2019). This could account, in part, for the observed differences in susceptibility to inflammation in the *Prevotella*-dominated microbiome of elderly individuals (Ticinesi et al, 2017).

Following these evidence, we addressed whether modulation of the microbiota composition through antibiotics (Khan et al, 2019; Tan et al, 2022) and germ-free mdx dystrophic model could foster DMD pathophysiology. GF condition enables the study of DMD progression from birth, whereas antibiotic treatment in adult mdx mice allows for the study of the role of microbiota in modulating muscle functionality and signaling pathways at specific times of disease. We showed that microbiota depletion in mdx is associated with a shift in fiber type toward an oxidative phenotype. Interestingly, ABX and GF conditions differentially affect change toward a slower MyHC profile, mirroring differences in the muscle adaptive responses to metabolic changes. This could be supported by findings in differential activation of AMPK-related pathways that mediate deregulation of muscle glucose uptake and enhanced fatty acid oxidation in mdx+ABX and GFmdx mice.

As such, the ABX-treated mdx and GFmdx mice displayed increased oxidative metabolism with reduced inflammation and fibrosis. The severe increase in the values of CPK in GFmdx revealed a more complex muscle-gut microbiota interaction. Our observations are in line with recent data describing the fundamental role of gut microbiota in the degradation of CPK (Clarke et al, 2014), suggesting the hypothesis that GFmdx could suffer from colonic dysfunctions as CPK modifications are associated with these pathologies (Qi et al, 2021; Vincent et al, 2018).

We thus verified whether correction of dysbiotic *Prevotella*-enriched microbiota of mdx can induce substantial modifications in skeletal muscle physiology and function. Intestinal Prevotella colonization results in metabolic changes in the microbiota, determining intestinal inflammation and systemic autoimmunity (Iljazovic et al, 2021). Since gnotobiotic colonization of GF mice with *Prevotella* species was shown to be ineffective (Geva-Zatorsky et al, 2017), we tested the effect of eubiotic microbiota in mdx. Our main finding was that eubiotic fecal transplantation in mdx mice was able to reduce gut-distal muscle immune responses with a parallel recovery of dystrophic muscle features. The increase of muscle carbohydrate metabolism and hepatic fatty acid oxidation could explain, at least in part, the recovered muscle metabolism and function of mdx mice. Other potentially interesting immunomodulatory activities were not reported previously, such as the augmentation of gut-derived regulatory CCR9 + CD4 + GITR+ subset in mdx mice receiving eubiotic microbiota. It will be interesting to see whether gut-derived regulatory CCR9 + CD4 + GITR+ cells will perform additional activities in skeletal muscles.

Multiple lines of investigation have revealed that DMD is characterized by primary and secondary features caused by dystrophin absence that occur in sequential phases. Notably, adaptive immunity is one of the secondary features of DMD, which proposes that an environmental factor triggers chronic muscle inflammation in the context of pre-existing innate immunity activation. Although a role for the gut microbiota has been clearly established in muscle homeostasis (Lahiri et al, 2019), it is not known if dysbiosis influences DMD. Our data suggest that residing gut microbial communities could be implicated in DMD progression, influencing clinical and phenotypic variability of dystrophic patients by modulating metabolic and immune response. All considering, the balance of microbiota composition is crucial to maintain correct muscle function, and the GF murine models retain both beneficial and deleterious effects (Hartmann et al, 2019). Despite the great research efforts, an effective cure for DMD is still missing. However, since patients are living longer, multidisciplinary management of DMD has become fundamental to prevent complications and alleviate disease progression. This study demonstrates that modulation of gut microbiota strain displacement presents interesting therapeutic opportunities to ameliorate DMD symptoms in patients. In addition, dietary interventions are the most effective and less-expensive strategies and represent a valuable co-adjuvant for DMD treatment by counteracting the damaging effects of chronic inflammation. Further characterization of microbiota changes in dystrophic patients should provide deeper insight into whether dysbiosis contributes to the progression of DMD.

## Methods

**Table:** Reagents and tools table

Reagent/resource	Reference or source	Identifier or catalog number
**Experimental models**		
C57BL (*M. musculus*)	Charles River	*C57BL/10*
mdx (*M. musculus*)	Charles River	*C57BL/10ScSn-Dmdmdx/J*
**Recombinant DNA**		
**Antibodies**		
CD3	Abcam	AB135372
Fibers type I	Developmental Studies Hybridoma Bank	BA-D5
Fibers type IIA	Developmental Studies Hybridoma Bank	Sc-71
Fibers type IIB	Developmental Studies Hybridoma Bank	BF-F3
PSMB5	Abcam	AB3330
PSMB8	Abcam	AB3329
PSMB9	Abcam	AB42987
β-actin	Sigma-Aldrich	a2066
LC3B	Sigma-Aldrich	L7543
TGFβ	Elabscience	e-ab-33090
TNFα	Elabscience	e-ab-40015
NF-kB	Santa Cruz Biotechnology	sc-514451
TRAF-6	Santa Cruz Biotechnology	sc-8409
RELb	Santa Cruz Biotechnology	sc-48366
PTX3	Abcam	AB90806
IL-6	Santa Cruz Biotechnology	sc-57315
VDAC1/Porin	Santa Cruz Biotechnology	sc-390996
PGC1α	Santa Cruz Biotechnology	sc-518038
FKHR-FOXO1	Santa Cruz Biotechnology	sc-374427
FKHRL1-FOXO3	Santa Cruz Biotechnology	sc-48348
IGF1	Santa Cruz Biotechnology	sc-9013
IGF2	Santa Cruz Biotechnology	sc-5622
TRPC-1	Santa Cruz Biotechnology	sc-20110
MTCO-1	Santa Cruz Biotechnology	sc-58347
IKK-I	Santa Cruz Biotechnology	sc-10760
AMPK-1α	Santa Cruz Biotechnology	sc-74461
GSK-3 αβ	Santa Cruz Biotechnology	sc-81496
TLR2	Biorbyt	orb229137
TLR4	Santa Cruz Biotechnology	sc-293072
vinculin	Invitrogen	MA5-11690
FGF21	Santa Cruz Biotechnology	sc-292879
MMP9	Abcam	ab38898
SIRT-1	Invitrogen	PA5-17074
ATG7	Sigma-Aldrich	sab4200304
GHSR	Elabscience	eab12471
GHRELIN	Invitrogen	pa1-1070
TOMM20	Abcam	AB186735
DRP1	Abcam	AB184247
SMAD3	Elabscience	e-ab-32921
SMAD2	Elabscience	e-ab-32916
P38	Elabscience	E-AB-32460
P62	Sigma-Aldrich	P0067
phosphoERK1-2	Elabscience	E-AB-20868
phosphoP38	Elabscience	E-AB-20949
phosphoSMAD2-3	Elabscience	E-AB-21-040
COX	Abcam	AB16056
IRS-1	Abcam	ab131487
AKT 1-2-3	Abcam	ab179463
PPARγ	Abcam	AB59256
IL-33	R&D	af3626
ERK 1-2	Abcam	ab54230
OPN	R&D	AF808
MyD88	Proteintech	23230-I-AP
HDAC1	Invitrogen	MA5-1807
HDAC2	Invitrogen	51-5100
VR-1	Santa Cruz Biotechnology	sc-12503
IGF1Rβ	Santa Cruz Biotechnology	sc-9038
IGF2R	Santa Cruz Biotechnology	sc-14413
CD45 PerCp	BD Bioscience	557235
CD4 Pe-Cy7	eBioscience	# 25-0042-82
CD4 Pacific Blue	BioLegend	100534
CD8 efluor 450	eBioscience	# 48-0088-42
CD44 FITC	BD Bioscience	553133
CD62L PE	eBioscience	# 12-0621-82
CD25 APC	eBioscience	# 17-0251-82
B220 APC-Cy7	eBioscience	# 25-0452-82
GITR Pe-Cy7	eBioscience	# 25-5874-82
CD3 FITC	eBioscience	# 11-0032-82
Foxp3 Alexa fluor 488	eBioscience	# 53-5773-82
F4/80 Pe-Cy7	eBioscience	# 25-4801-82
CD11b PE	eBioscience	# 12-0112-82
CD11c FITC	eBioscience	# 11-0114-82
IL-17 PE	eBioscience	# 12-7471-82
IFN-γ APC	eBioscience	# 17-7311-82
**Oligonucleotides and other sequence-based reagents**		
PCR primer	This study	Table 1
**Chemicals, enzymes, and other reagents**		
Vancomycin	Thermo Fisher	J62790.03
Ampicillin	Thermo Fisher	11593027
Metronidazole	Thermo Fisher	210345000
Hematoxylin Weigert’s Iron Part A	Bio-Optica	05-B06008A
Hematoxylin Weigert’s Iron Part B	Bio-Optica	05-B06008B
Phosphomolybdic acid	Bio-Optica	05-M05003
Aniline Blue solution	Bio-Optica	05-B10006
Glacial acetic acid	Carlo Erba	200-580-7
Xylene	Sigma-Aldrich	534056
DPX reagent	VWR International	06522
Prolong Gold® Antifade Reagent with DAPI	Thermo Fisher	P36931
Reverse Transcriptase Kit	ThermoFisher Scientific	18090200
nitrocellulose membranes	Bio-Rad Laboratories	1620113
70μm nylon strainers	BD Biosciences	352350
**Software**		
The Ribosomal Database Project (RDP) Classifier	V2.11	
Greengenes database	release 13_08	
iPATH 3	https://pathways.embl.de	
PANTHER Enrichment Test	release 16.0	
ImageJ Software (NIH)	release v1.53n	
GraphPad Prism^TM^	release 9.2.0	
FlowJo software	release v9	
**Other**		
cryostat	Leica Microsystems	CM 1900
indium–tin oxide (ITO) slides	Bruker Daltonik	8259387
0.2-mm nozzle caliber airbrush	Procon Boy FWA Platinum	GUN-PS-270
CP-Sil 8 CB fused silica capillary GC column	Agilent	CP7599
Microdissector	Leica Microsystems	CTR6000
iMScope Microscope	Shimadzu	TRIO Mass
MetaboPrep kit	Theoreo	
Gas chromatograph and single quadrupole mass spectrometer	Shimadzu	GC-2010 Plus

### Animal ethics statement

Procedures involving living animals were conformed to Italian law (D.L.vo 116/92 and approved by local ethics committees, according to ARRIVE guidelines. This work was authorized by the Ministry of Health and the Local University of Milan Committee, authorization number 859/2017-PR (5247B.35, 10/07/2017). Two-month-old C57BL/10 and mdx male mice from Charles River were maintained at the Policlinico Hospital animal facility. All animals were housed in ventilated cages in a 12 h light/dark cycle, with free access to water and standard autoclaved chow. Upon arrival, mice were allowed to acclimate to the animal facility environment for at least 4 weeks, and analysis was performed on 3-month-old animals. Food intake was measured. No food intake differences were observed between C57BL/10 and mdx mice.

Individual mice were placed in empty autoclaved cages and monitored for defecation: stool samples were collected with autoclaved toothpicks. For the microbiota depletion experiments, 3-month-old mice were orally gavaged with a mix of antibiotics (ABX) containing Vancomycin (1.25 mg), ampicillin (2.5 mg), and metronidazole (1.25 mg) (all from Thermofisher) in 200 µl water for 4 weeks. For the Fecal Microbiota Transplantation (FMT) experiments, 3-month-old mdx mice were pre-treated with the ABX cocktail in 200 µl water per mouse by oral gavage for 7 days to promote a more efficient bacterial colonization (Ji et al, 2017) and then transplanted with feces from C57Bl/10 (ABX-mdx^FMT_C57Bl^), as previously described (Burrello et al, 2018; Strati et al, 2021). Feces collected from donor mice were diluted in PBS (1:10 w/v), briefly centrifuged to remove large debris, and 200 μl of this fecal slurry was given to recipients daily for 5 days by oral gavage.

Randomization within blocks was performed to allocate the animals to different experimental procedures. To avoid the effects of our treatments on mice being overestimated, thus diminishing the reliability of our results, the laboratory members who analyzed the mice were blinded regarding the treatment(s) that the animals received during all the experimental procedures. Animals that eventually suffered from clinical complications during each treatment (enhancement of stress, motor impairments) were excluded from the experimental plan.

### GFmdx derivation

We participated in the EC Horizon 2020-funded INFRAFRONTIER2020 project (2017–2020), to obtain mouse axenic service implemented as a Trans-national Access activity. INFRAFRONTIER is the European Research Infrastructure for phenotyping, archiving, and distribution of model mammalian genomes by the European Mouse Mutant Archive (EMMA), providing access to tools and data for biomedical research (www.infrafrontier.eu). Through the “INFRAFRONTIER2020 project and microbiome research”, in collaboration with the Gnoto/Axenic Facility of the Instituto Gulbenkian de Ciência—partner and founding member of the ECGnoto network http://www.ecgnoto.eu—we generated the GFmdx. A detailed procedure is described below.

### Standard operative procedure (SOP) for generating germ-free (Axenic) mice using cesarean section rederivation

#### Equipment

Sterile isolator and set up for rearing germ-free mice.

Transfer chamber compatible with the isolator.

Autoclaved water inside the transfer chamber.

#### Surgical equipment

Medroxiprogesterone acetate (150 mg/ml, Pfizer)

VirkonS, 1% solution at Room Temperature (RT) (Antec Int. Ltd.).

#### Method

Ensure availability of isolator-reared, germ-free surrogate mother with newborn pups (< 5 days old) at day 19 of procedure (see below).

Day −2—Set up the relevant mating of the foster strain inside the recipient isolator (usually on Sunday).

Day −1—Check for mating plugs inside the isolator and identify the foster females.

Day 0—Check for mating plugs inside the isolator and identify the foster females. If more than two plugs between day −1 and day 0, set up the relevant mating of the mouse strain to be converted to germ-free status (usually on Wednesday).

Day 1—Check for mating plugs inside and outside the isolator. Identify the foster and donor female(s) for the experiment.

Day 2—Check for mating plugs inside and outside the isolator. Identify the foster and donor female(s) for the experiment. Separate females from males (if some remain without a plug) inside the isolator.

Day 3—Check for mating plugs outside the isolator. Identify the donor female(s) for the experiment. Separate females from males (if some remain without a plug), from the strain to be converted to germ-free.

Day 18—Check pregnancies inside and outside the isolators. Give pregnant donor female(s) from day 1, at 17.5 days post coitus (dpc), a subcutaneous injection of medroxiprogesterone acetate (5 mg/0.1 ml).

Day 19—Carefully following the SOP for isolator entry procedures, transfer the sterile instruments and supplies required for surgery into the isolator in which the surrogate female(s) are housed. Prepare the hysterectomy suite/surgical transfer chamber: fill up the reservoir with 1% VirkonS, sterilize the surgical compartment, and ventilate it overnight. Give pregnant donor female(s) from day 2, at 17.5 dpc, a subcutaneous injection of medroxiprogesterone acetate (5 mg/0.1 ml).

Day 20—Give pregnant donor female(s) from day 3, at 17.5 dpc, a subcutaneous injection of medroxiprogesterone acetate (5 mg/0.1 ml). Transfer water, paper towels, and surgical instruments from the isolator to the sterilized compartment of the transfer chamber. Working in the non-sterile compartment of the surgical transfer chamber or the place where the animals are allocated, sacrifice the donor female by cervical dislocation and submerge the whole animal in the 1% VirkonS solution for 1 min. Use sterile scissors to open the abdomen. Clamp the top of each uterine horn and the base of the uterus close to the cervix, with Mosquito scissors. Cut out the “uterine package” and place it in the transfer chamber reservoir filled with 1% VirkonS for 1 min. This procedure can be performed for a max of 2 females at the same time. Inside the sterile compartment of the transfer chamber, rinse the “uterine package” with sterile water to remove the VirkonS (200 ml minimal volume of water). On top of a heating pad at 37 °C, open the “uterine package” with scissors and take out the pups, taking care do not cut the umbilical cord. After removing the pup from the placenta, gently pull the umbilical cord with your forceps. Stimulate breathing of the pups while cleaning them with a dry paper towel. When pups are breathing normally and have gained a healthy skin color, transfer them to the isolator housing the foster mother. Gently rub the pups with bedding material from the foster mother’s cage. Leave them mixed with the bedding 1 or 2 min. Remove some of the original pups so that the foster mother has the same number of pups to feed. If some pups from the foster mother remain in the cage, mix the adopted ones with them (clean the bedding). Check for adoption not earlier than 24 h after transfer. Monitor the microbiological status of the isolator and the animals it houses 3 weeks after transfer.

Day 21/22—repeat step 20 for pregnant donor females on days 2 and 3, if necessary.

### FACS analysis

Murine lamina propria mononuclear cells (LPMC) were isolated as described in (Weigmann et al, 2007). Briefly, colonic lamina propria mononuclear cells were isolated via incubation with 5 mM EDTA at 37 °C for 30 min, followed by mechanical disruption with GentleMACS (Miltenyi Biotec). After filtration with 100-μm and 70-μm nylon strainers (BD), the LPMC were counted and stained for immunophenotyping.

Pooled muscle from the leg (vastus medialis, vastus lateralis, rectus femoris, biceps femoris, adductors, and gastrocnemius) and spleen of ABX-treated mdx, GFmdx, ABX-mdx^FMT_C57Bl^, and age-matched untreated mdx or C57Bl mice were minced slightly to remove blood-trapped vessels and dispersed with scissors to increase total surface area, to enhance the efficiency of digestion while shortening the time required for this procedure. Tissues were washed in PBS and then digested at 37 °C with 0.2 mg/ml Liberase in DMEM culture medium. Undigested tissues were mashed with a plunger through the filters and washed with DMEM with serum. Then, they were filtered through a 70-µm filter, placed on Histopaque 1077 gradient, and centrifuged at 400× *g* for 45 min. We harvested the cells at the interface, washed them twice with PBS, and then used them for flow cytometry analysis. Cells obtained from the muscle, spleen, and colon lamina propria tissues from the same mice were evaluated for the expression of different immunological subpopulations. Cells were multiple-labeled with different groups of antibodies to recognize specific subpopulations (for muscle: CD45 PerCp, CD4 Pe-Cy7 and Pacific Blue, CD8 efluor 450, CD44 FITC, CD62L PE, CD25 APC, B220 APC-Cy7, GITR Pe-Cy7, CD3 FITC. For spleen: CD4 Pe-Cy7 and Pacific Blue, CD8 efluor 450, CD44 FITC, CD62L PE, Foxp3 Alexa fluor 488, CD25 APC, F4/80 Pe-Cy7, CD11b PE, CD11c FITC, IL-17 PE, IFN-γ APC. For GI: CD45 PerCp, CD11b PE, Lys). All the antibodies were purchased from eBioscience (San Diego, USA), except for CD45 PerCp, CD44 FITC obtained from BD (New Jersey, USA), and CD4 Pacific Blue from BioLegend (San Diego, USA). For FACS characterization, data were acquired with the BD Canto II machine and analyzed with FlowJo 9 software. Each analysis included at least 5–10 × 10^4^ events for each gate.

### Serum analysis

CPK, ALT, AST, and GLUC3 analysis were performed on serum samples of ABX-treated mdx, GFmdx, ABX-mdx^FMT_C57Bl^, and untreated mdx mice with CPK/ALT/AST/GLUC3 kit (Cobas), according to the manufacturer’s instructions.

### Analysis of tetanic force

Tetanic force of the Tibialis Anterior (TA) muscle was determined as described in (Farini et al, 2016), normalized to muscle cross-sectional area and expressed as kN/m^2^.

### Histological analysis

Colon tissues were collected from 3 m C57Bl/10 and mdx, frozen in liquid-nitrogen cooled isopentane, and cut on a cryostat into 10-µm slices. H&E staining was performed as in (Farini et al, 2021). TA muscle tissues were collected from ABX-treated mdx, GFmdx, ABX-mdx^FMT_C57Bl^ and untreated mdx mice, frozen in liquid-nitrogen cooled isopentane and cut on a cryostat into 10 µm. Gömöri trichrome staining was performed to evaluate the morphology and the percentage of fibrosis. Adjacent sections were stained with H&E. Frozen sections were brought to RT and placed in preheated Bouin’s Fluid (BF) at 56 °C for 15 min. Equal volumes of Hematoxylin Weigert’s Iron Part A and B (Bio-Optica, Milan S.p.A., Italy) were applied to tissue sections for 5 min. Then, acid alcohol solution (0,5%) was applied to sections for 10 s, to stain cytoplasm, followed by Acid Fuchsin solution (Bio-Optica, Milan S.p.A., Italy) diluted 1:2 in deionized water for 5 min. Tissue sections were incubated with Phosphomolybdic acid (Bio-Optica, Milan S.p.A., Italy) for 5 min to block the staining of all tissue components other than connective tissue fibers. Then, slides were incubated with Aniline Blue solution (Bio-Optica, Milan S.p.A., Italy) for 5 min to stain collagen fibers. Finally, slides were washed in deionized water combined with 1% glacial acetic acid (Carlo Erba, Milan, Italy) and incubated for 30 s in 100% ethanol solution, for dehydration. 100% Xylene (Sigma-Aldrich, USA) for 1 min before mounting with DPX reagent (VWR International, USA) and coverslips. Frozen sections were characterized by immunofluorescence staining. Slides were fixed with 4% paraformaldehyde for 10 min, permeabilized with 0.3% Triton X-100 for 15 min and incubated with 10% donkey serum to block non-specific binding for 1 h and then incubated with the primary antibodies (overnight at 4 °C) diluted in blocking solution. Fluorochrome-conjugated secondary antibodies were diluted in PBS and added for 1 h at room temperature. Primary antibodies were used at the following dilutions: CD3 1:50 (AB135372, Abcam, UK); fibers type I 1:50 (BA-D5, Developmental Studies Hybridoma Bank, Douglas Houston); fibers type IIA 1:50 (sc-71, Developmental Studies Hybridoma Bank); fibers type IIB 1:50 (BF-F3, Developmental Studies Hybridoma Bank). Slides were then mounted with Prolong Gold® Antifade Reagent with DAPI (Thermo Fisher, Carlsbad, CA). A Leica DMi8 fluorescence microscope was used for acquiring images. Histological identification of slow/type I, fast fatigue-resistant/type Iia, and fast fatigable/type Iib fibers was performed by staining for either myosin ATPases or oxidative enzyme capacity (succinate dehydrogenase, SDH). Enzymatic activity of SDH was assayed by placing the slides in SDH incubating solution, containing sodium succinate as a substrate and nitro-blue tetrazolium (NBT) for visualization of the reaction for 1 h at 37 °C. At first, slides were incubated for 10 s in 30–60–90–60–30% acetone solution and, then, for 30 s in 80–90–100% ethanol solution for dehydration. Finally, 100% Xylene (Sigma-Aldrich, USA) for 1 min before mounting with DPX reagent (VWR International, USA) and coverslips. For Gömöri trichrome and SDH staining, images were captured by a Leica microdissector (CTR6000).

### Imaging mass spectrometry

Colon tissues of 3-month-old mdx mice were frozen for preparation of cryosections (thickness of 10 μm) with the use of a cryostat (CM 1900; Leica Microsystems, Wetzlar, Germany). For imaging mass spectrometry, the sections were thaw-mounted on indium–tin oxide (ITO) slides (Bruker Daltonik, Bremen, Germany), dried in silica gel–containing plastic tubes, and then sprayed with 9-aminoacridine (5 mg in 4 ml of 80% ethanol) with the use of a 0.2-mm nozzle caliber airbrush (Procon Boy FWA Platinum; Mr Hobby, Tokyo, Japan) for matrix-assisted laser desorption-ionization (MALDI) imaging mass spectrometry in positive-ion mode. Adjacent sections were stained with H&E. Imaging mass spectrometry was performed with iMScope TRIO Mass Microscope (Shimadzu, Kyoto, Japan). MALDI mass spectra were acquired with a laser diameter of 50 μm, 200 shots/spot, scanning pitch of 20 μm, and scanning *m/z* range of 615–931. Regions of tissue samples exposed to the laser radiation were determined by light and fluorescence microscopic observations. For each lipid, the mean intensity was measured by ImageJ Software at 12 positions (sample area of 100 × 100 μm^2^) throughout the colon images.

### Qualitative (RT-qPCR) experiments

Total RNA was extracted from TA muscle of ABX-treated, GF, and age-matched untreated mdx or C57Bl mice, and cDNA was generated using the Reverse Transcriptase Kit (ThermoFisher Scientific, CA, USA). We quantified the expression of genes through the SYBR-Green method. All the samples were tested in duplicate, and the threshold cycles (Ct) of target genes were normalized against the housekeeping gene, β-actin. Relative transcript levels were calculated from the Ct values as X = 2^−ΔΔct^ where X is the fold difference in amount of target gene versus β-actin and ΔCt=Ct_target_ − Ct_β-actin_. The efficiency of primers used was calculated between 95.2% and 98.9%. The sequence of primers used is listed in Table 1.

**Table 1:** List of primers (5’➔3’) for RT-qPCR.

Actn3 f AATCGCCAACGTTAACAAGG
Actn3 r AGTGTTCAGGTTTCCGATGG
Chrnd f TCGTCGCAAACCGCTCTT
Chrnd r GATGGCCAGCGAGGTGAT
Col1a f CCTCAGGGTATTGCTGGACAAC
Col1a r CAGAAGGACCTTGTTTGCCAGG
Col3a-f CCTTAACATGTGTCTTTAAAGCCC
Col3a-r AAATGCTTTTAAAGGTGCTTCTCT
CoxVa-f TTGATGCCTGGGAATTGCGTAAAG
CoxVa-r AACAACCTCCAAGATGCGAACAG
CoxVIIb-f TTTCAGGACGCTTTGCAAGG
CoxVIIb-r TGCTTCGAACTTGGAGACGG
CytC f CATCTCAACGGCTTATTATGACTTT
CytC r GCTAACCACCAGGAGGCAACTGT
Ldh f TATCTTAATGAAGGACTTGGCGGATGAG
Ldh r GGAGTTCGCAGTTACACAGTAGTC
matp2a1-f TGTTTGTCCTATTTCGGGGTG
matp2a1-r AATCCGCACAAGCAGGTCTTC
Mcad f TAC GGC ACA AAA GAA CAG ATC G
Mcad r CAG GCT CTG TCA TGG CTA TGG
mFoxP3-f TCAAGTACCACAATATGCGA
mFoxP3-r GATTTCATTGAGTGTCCTCTG
mGPx1-f AGTTCGGACATCAGGAGAATGGCA
mGPx1-r TCACCATTCACCTCGCACTTCTCA
miNOS-f CTCACTGGGACAGCACAGAA
miNOS-r GGCCTTGTGGTGAAGAGTGT
mMurF1-f CAGAGGCAGTTGGATCGTCTATG
mMurF1-r TGAGGCAGAGTCTCTCTATGT
mMYHCs12-r TTCACCTGGGACTCAGCAATG
mMYHCsl2-f AAGCTGAGGAGGCTGAGGAAC
mNRF1-f GGCACTGTCTCACTTATCCAGGTT
mNRF1-r CAGCCACGGCAGAATAATTCA
mp62-f AGGCGCACTACCGCGAT
mp62-r CGTCACTGGAAAAGGCAACC
mpdk4-f GTCTCAATAGTGTCACCTGTGTAA
mpdk4-r CCTGGGCATTTAGCATCTATCT
mPGC1α-f GCTAAACGACTCCGAGAACAA
mPGC1α-r ACTGACCCAAACATCATACCC
mPPARα-f TGATTGGTTCCAGGCAATTAGA
mPPARα-r CACTCGTACAGTCAGTTCAGTC
Mrf4 f GCACGCAGTGCTTCTTC
Mrf4 r CATGCTGCTGTCTGAAGGTC
mRORγt-f GACTGACAATCAGCAGGGATAA
mRORγt-r GGGAAATACAATGAGGTATTGAAAGG
mTbet-f GATCATCACTAAGCAAGGAC
mTbet-r ACATCCACAAACATCCTGTA
Myf5 f CTGCTCTGAGCCACCAG
Myf5 r GACAGGGCTGTTACATTCAGG
MyHC-IIb f CAAGAGACAAGCTGAAGAGGCT
MyHC-IIb r GATATACAGGACAGTGACAAAGAACT
MyoD f AGCACTACAGTGGCGACTA
MyoD r GGCCGCTGTAATCCATCA
Myogenin f CCTTGCTCAGCTCCCTCA
Myogenin r TGGGAGTTGCATTCACTGG
Myogl f GAGGGAGCTGGTGTCAACAG
Myogl r CTTGCAAAACCACACTGCTC
Pax7 f AAAAAACCCTTTCCCTTCCTACA
Pax7 r AGCATGGGTAGATGGCACACT
Tnnt1 f AAGGGGAGCGTGTGGATTTTG
Tnnt1 r TCCTCCTTTTTCCGCTGTTCA
β-actin f GGCTGTATTCCCCTCCATCG
β-actin r CCAGTTGGTAACAATGCCATGT

### WB analysis

TA skeletal muscles and colonic tissues were isolated from ABX-treated mdx, GFmdx, ABX-mdx^FMT_C57Bl^, and age-matched untreated mdx or C57Bl mice, and total proteins were obtained as in (Parolini et al, 2009). Samples were resolved on polyacrylamide gels (ranging from 6 to 14%) and transferred to nitrocellulose membranes (Bio-Rad Laboratories, CA, USA). Filters were incubated overnight with following antibodies: PSMB5 (1:500, AB3330, Abcam); PSMB8 (1:500, AB3329, Abcam); PSMB9 (1:500, AB42987, Abcam); β-actin (1:500, a2066, Sigma-Aldrich); LC3B (1:500, L7543, Sigma-Aldrich); TGFβ (1:500, e-ab-33090, Elabscience); TNFα (1:500, e-ab-40015, Elabscience); NF-kB (1:500, sc-514451, Santa Cruz Biotechnology, SCB); TRAF-6 (1:500, sc-8409, SCB); RELb (1:500, sc-48366, SCB); PTX3 (1:500, AB90806, Abcam); IL-6 (1:500, sc-57315, SCB); VDAC1/Porin (1:500, sc-390996, SCB); PGC1α (1:500, sc-518038, SCB); FKHR-FOXO1 (1:500, sc-374427, SCB); FKHRL1-FOXO3 (1:500, sc-48348, SCB); IGF1 (1:500, sc-9013, SCB); IGF2 (1:500, sc-5622, SCB); TRPC-1 (1:500, sc-20110, SCB); MTCO-1 (1:500, sc-58347, SCB); IKK-I (1:500, sc-10760, SCB); AMPK-1α (1:500, sc-74461, SCB); GSK-3 αβ (1ː500, sc-81496, SCB); TLR2 (1500, orb229137, Biorbyt): TLR4 (1:500, sc-293072, SCB); vinculin (1:500, MA5-11690, Invitrogen); FGF21 (1:500, sc-292879, SCB); MMP9 (1:500, ab38898, Abcam); SIRT-1 (1:500, PA5-17074, Invitrogen); ATG7 (1:500, sab4200304, Sigma-Aldrich); GHSR (1:500, eab12471, Elabscience); GHRELIN (1:500, pa1-1070, Invitrogen); TOMM20 (1:500, AB186735, Abcam); DRP1 (1:500, AB184247, Abcam); SMAD3 (1:500, e-ab-32921, Elabscience); SMAD2 (1:500, e-ab-32916, Elabscience); P38 (1:500, E-AB-32460, Elabscience); P62 (1:500, P0067, Sigma-Aldrich); phosphoERK1-2 (1:500, E-AB-20868, Elabscience); phosphoP38 (1:500, E-AB-20949, Elabscience); phosphoSMAD2-3 (1:500, E-AB-21-040, Elabscience); COX IV (1:500, AB16056, Abcam): IRS-1 (1:500, ab131487, Abcam); AKT 1-2-3 (1:500, ab179463, Abcam); PPARγ (1:500, AB59256, Abcam); IL-33 (1:500, af3626, R&D); ERK 1-2 (1:500, ab54230, Abcam); OPN (1:500, AF808, R&D); MyD88 (1:500, 23230-I-AP, Proteintech); HDAC1 (1:500, MA5-1807, Invitrogen); HDAC2 (1:500, 51-5100, Invitrogen); VR-1 (1:500, sc-12503, SCB); IGF1Rβ (1:500, sc-9038, SCB); IGF2R (1:500, sc-14413, SCB). Filters were detected with peroxidase-conjugated secondary antibodies (Agilent Technologies, CA, USA) and developed by ECL (Amersham Biosciences, UK).

### Microbiota analysis

DNA extraction, 16S rRNA gene amplification, purification, library preparation, and pair-end sequencing on the Illumina MiSeq platform were performed as described in (Burrello et al, 2019). Reads were pre-processed using the MICCA pipeline (v.1.7.0) (https://micca.readthedocs.io/en/latest/index.html) (Albanese et al, 2015). Forward and reverse primers trimming and quality filtering were performed using micca trim and micca filter, respectively. Filtered sequences were denoised using the UNOISE (Edgar, 2016) algorithm implemented in micca otu to determine true biological sequences at the single-nucleotide resolution by generating amplicon sequence variants (ASVs). Bacterial ASVs were taxonomically classified using Micca classify and the Ribosomal Database Project (RDP) Classifier v2.11 (Wang et al, 2007). Multiple sequence alignment (MSA) of 16S sequences was performed using the Nearest Alignment Space Termination (NAST) algorithm (DeSantis et al, 2006b) implemented in micca msa with the template alignment clustered at 97% similarity of the Greengenes database (DeSantis et al, 2006a) (release 13_08). Phylogenetic trees were inferred using the Micca tree (Price et al, 2010). Sampling heterogeneity was reduced by rarefying samples at the depth of the less abundant sample using micca tablerare. Alpha (within-sample richness) and beta-diversity (between-sample dissimilarity) estimates were computed using the phyloseq R package (McMurdie and Holmes, 2013). Permutational multivariate analysis of variance (PERMANOVA) test was performed using the adonis function in the R package vegan with 999 permutations. Differential abundance testing was carried out using the R package DESeq2 (Love et al, 2014) using the non-rarefied data (McMurdie and Holmes, 2014). *P* values were False Discovery Rate corrected using the Benjamini–Hochberg procedure implemented in DESeq2. Random Forest (Breiman, 2001) analyses of 16S rRNA gene sequencing data were performed using the random Forest R package; permutation tests with 1000 permutations were performed to assess model significance (Murphy et al, 2010). Spearman’s correlation tests were computed using the psych R package (Revelle and Wilt, 2013). Prediction of functional metagenomic content was inferred by using Piphillin (Narayan et al, 2020) with the reference curated databases BioCyc (Caspi et al, 2014) and Kyoto Encyclopedia of Genes and Genomes (KEGG) (Kanehisa and Goto, 2000). Metabolic pathway maps were visualized using iPATH 3 (https://pathways.embl.de/) (Darzi et al, 2018).

### RNA-seq analysis

Library Preparation and DNA Sequencing: 150–300 ng of the total RNA determined by InvitrogenTM QubitTM high-sensitivity spectrofluorometric measurement was poly-A selected and reverse transcribed using Illumina’s TruSeq stranded mRNA library preparation kit. Each sample was fitted with one of 96 adapters containing a different 8-base molecular barcode for high-level multiplexing. After 15 cycles of PCR amplification, completed libraries were sequenced on an Illumina NovaSeqTM 6000, generating 20 million or more high-quality 100-base long paired-end reads per sample. RNA-Seq Analysis: A quality control check on the fastq files was performed using FastQC. Upon passing basic quality metrics, the reads were trimmed to remove adapters and low-quality reads using default parameters in Trimmomatic1 (Bolger et al, 2014). Alignment, Transcript Abundance and Differential Gene Expression Analysis: The trimmed reads were then mapped to a reference genome using default parameters with the strandness (R for single-end and RF for paired-end) option in Hisat22 (Kim et al, 2015). In the next step, transcript/gene abundance was determined using kallisto3 (Bray et al, 2016). We first created a transcriptome index in Kallisto using Ensembl cDNA sequences for the reference genome. This index was then used to quantify transcript abundance in raw counts and transcript per million (TPM). Fold-changes between groups were calculated using EdgeR from the Bioconductor package (Robinson et al, 2010). PCA analysis on differentially expressed genes was performed using ClustVis (Metsalu and Vilo, 2015). Gene ontology (GO) analysis was conducted, submitting gene lists to the PANTHER Enrichment Test (release 16.0), a built-in analytical tool in the AmiGO2 software suite by the Gene Ontology consortium (Mi et al, 2021). GO analyses were conducted on the GO Ontology database (version 2021-05-01), using all genes in the Mus musculus database as a reference list and the GO Biological Process Complete as an annotation dataset. Significantly enriched GO terms were identified by adjusted *P* value < 0.05. GSEA was performed via dedicated software (release 4.2.3) by the Molecular Signatures Database (MSigDB). The “Hallmark” annotated gene set collection was used for analysis of ranked gene lists.

### Metabolome analysis

To extract the metabolome from the GI tissues of ABX-treated and age-matched untreated mdx or C57Bl mice, we used the MetaboPrep kit (Theoreo, Montecorvino Pugliano, SA) as in (Strati et al, 2021; Troisi et al, 2018) according to the manufacturer’s protocol. Analysis was conducted in gas chromatography coupled with mass spectrometry (GC-2010 Plus gas chromatograph and 2010 Plus single quadrupole mass spectrometer; Shimadzu Corp., Kyoto, Japan). Chromatographic separation was achieved as previously reported using a 30 m 0.25 mm CP-Sil 8 CB fused silica capillary GC column with 1.00-μm film thickness from Agilent (J&W Scientific, Folsom, CA, USA), with helium as carrier gas. Untargeted metabolites were identified by comparing the mass spectrum of each peak with the NIST library collection (NIST, Gaithersburg, MD, USA). To identify metabolites, the linear index difference max tolerance was set at 50, while the minimum matching for the NIST library search was set at 85%. According to the MSI level 1 standard (Sumner et al, 2007), the relevant putative metabolites were further confirmed using an independent analytical standard analysis. The normalization procedures consisted of data transformation and scaling. Statistical analyses were conducted on transformed (og transformation) and autoscaled (mean-centered and divided by the standard deviation of each variable) data. Partial least square discriminant analysis (PLS-DA) was performed on an internal standard peak area normalized chromatogram using R. Classification and cross-validation were performed using the wrapper function included in the caret package. A permutation test was performed to assess the significance of class discrimination. Variable importance in projection (VIP) scores were calculated for each component. For each relevant metabolite, the Mouse Metabolome Database (MMDB) ID number was determined. Metabolic pathways associated with these metabolites were analyzed using the MetScape application (Karnovsky et al, 2012). Metabolic pathway involvement was also evaluated using the MetPa tool (Xia and Wishart, 2010).

### Image quantification

Histological images were captured by a Leica microdissector, a fluorescent microscope, and a confocal microscope. Quantitative analyses were performed by ImageJ Software (NIH). The threshold color Plug-in of ImageJ Software was used to quantify the Gömöri trichrome staining as a percentage of area over a fixed grid area. For IF quantification, confocal acquisition of *n* = 12 muscle cross-sections for distinct TA muscles was obtained from each experimental animal used for each protocol. Data were analyzed by GraphPad Prism^TM^ and expressed as means ± SD.

### Statistics

To determine the significance of the variation of cellular concentration throughout the time, we used the linear regression for repeated measures. To compare multiple-group means, one-way ANOVA followed by Tukey’s multiple comparison test or nonparametric test followed by Kruskal–Wallis test were used to determine significance (*P < 0.05, **P < 0.01, ***P < 0.001; ****P < 0.0001). To compare two groups, Student’s *t* test was applied assuming equal variances (*P < 0.05, **P < 0.01, ***P < 0.001; ****P < 0.0001). Sample size was determined by using a sample-size calculator freely available on the internet. All the samples that did not meet quality control standards due to the presence of contaminants for RNA or problems in freezing procedures for histological analysis were excluded. The analysis of the Alpha-diversity index to evaluate microbiota richness was based on the Wilcoxon rank-sum test on row data.

## Supplementary information

Appendix

Data Set EV1

Data Set EV2

Data Set EV3

Data Set EV4

Source data Fig. 1

Source data Fig. 2

Source data Fig. 3

Source data Fig. 4

Source data Fig. 5

Source data Fig. 6

Source data Fig. 6E

Source data Fig. 7

Figure EV Source Data

Expanded View Figures

## Data Availability

All data are available in the main text or the supplementary materials. The RNAseq data have been deposited in the GEO Database (accession number: GSE218370). The source data of this paper are collected in the following database record: biostudies:S-SCDT-10_1038-S44321-026-00445-1.
