# Supplementary material for: Microbiota dysbiosis influences immune system and muscle pathophysiology of dystrophin deficient mice
Source: EMBO Mol Med. 2026 Jun 3;18(7):2979–3008. doi: 10.1038/s44321-026-00445-1 (PMC13365508; doi:10.1038/s44321-026-00445-1)
Supplement: Supplementary file 1 — Appendix [file 44321_2026_445_MOESM1_ESM.pdf]

Table of content

Appendix Table S1: Table with the exact p-values in Manuscript

Figure	Experiment		Animal models	p-value
Figure 1C	Mucus layer thickness		3m C5Bl vs 3m mdx	<0,0001
	Crypt length		3m C5Bl vs 3m mdx	<0,0001
Figure 1E	Phosphatidylcholines (PC) and Lysophosphatidylcholines (LysoPC)	Lyso PC 18:0 vs. Lyso PC 16:0		0,0015
		Lyso PC 18:0 vs. PC 34:2		<0,0001
		Lyso PC 18:0 vs. PC 36:2		<0,0001
		Lyso PC 16:0 vs. PC 34:2		0,0003
		Lyso PC 16:0 vs. PC 36:2		<0,0001
		PC 34:2 vs. PC 36:2		0,0294
Figure 1F	WB analysis of colon	PSMB8	3m C5Bl vs 3m mdx	0,0089
		RelB	3m C5Bl vs 3m mdx	0,0071
		PTX3	3m C5Bl vs 3m mdx	0,0168
		TLR2	3m C5Bl vs 3m mdx	0,0012
Figure 3B	Predicted gene content	propionyl-CoA:succinate CoA transferase	3m C5Bl vs 3m mdx	0,0062
		propionate CoA-transferase	3m C5Bl vs 3m mdx	0,0006
		butyryl-CoA:acetate CoA-transferase	3m C5Bl vs 3m mdx	0,0005
Figure 4E	Total SCFA		3m mdx vs 3m mdx+ABX	0,0001
	Acid acetic		3m mdx vs 3m mdx+ABX	0,0027
Figure 5B	% of splenic CD4+ T-cells	Effector	3m C57Bl vs. 3m mdx	0,043
			3m C57Bl vs. 3m mdx + ABX	0,0324
Figure 5D	% of muscle CD4+ T-cells	Effector	3m C57Bl vs. 3m mdx	0,0024
			3m C57Bl vs. 3m mdx + ABX	0,0381
Figure 5F	% of splenic lymphocytes	Tregs	3m mdx vs. 3m mdx + ABX	0,0054

		CD4+ T-cells	3m C57Bl vs. 3m mdx	0,0181
			3m C57Bl vs. 3m mdx + ABX	<0,0001
			3m mdx vs. 3m mdx + ABX	<0,0001
		CD8+ T-cells	3m C57Bl vs. 3m mdx + ABX	0,0019
			3m mdx vs. 3m mdx + ABX	0,0001
Figure 5G	% of CD45+ cells		3m C57Bl vs. 3m mdx	0,0111
			3m C57Bl vs. 3m mdx + ABX	0,9258
			3m mdx vs. 3m mdx + ABX	0,0073
Figure 5I	% of CD3+ cells/mm^2		3m C57Bl vs. 3m mdx	0,0007

Figure 6D	RT-qPCR experiments	pax7	3m mdx vs. 3m GFmdx	0,0053
		myf5	3m mdx vs. 3m mdx + ABX	0,0086
		actn3	C57Bl vs. 3m mdx + ABX	0,0015
			3m mdx + ABX vs. 3m GFmdx	0,0143
		myod	C57Bl vs. 3m mdx	0,0273
			3m mdx vs. 3m mdx + ABX	0,0046
			3m mdx vs. 3m GFmdx	0,0247
		myogenin	C57Bl vs. 3m GFmdx	0,0012
		mrf4	C57Bl vs. 3m GFmdx	0,0075
		murf1	C57Bl vs. 3m mdx	0,0086
			C57Bl vs. 3m mdx + ABX	0,0253
			C57Bl vs. 3m GFmdx	0,0002
		tnnt1	3m mdx vs. 3m GFmdx	0,0143
Figure 6E	Myofiber area		3m c57bl vs. 3m mdx	<0,0001

			3m c57bl vs. 3m mdx + ABX	<0,0001
			3m c57bl vs. 3m GFmdx	<0,0001
			3m mdx vs. 3m mdx + ABX	<0,0001
			3m mdx vs. 3m GFmdx	<0,0001
			3m mdx + ABX vs. 3m GFmdx	<0,0001
Figure 6F	% of fibrosis		3m c57bl vs. 3m mdx	<0,0001
			3m c57bl vs. 3m mdx + ABX	<0,0001
			3m c57bl vs. 3m GFmdx	<0,0001
			3m mdx vs. 3m mdx + ABX	<0,0001
			3m mdx vs. 3m GFmdx	0,0204
			3m mdx + ABX vs. 3m GFmdx	<0,0001
Figure 6H	Myosin isoform	IIA Fibers	3m C57Bl vs. 3m mdx	0,0029
			3m C57Bl vs. 3m GFmdx	0,0067
			3m mdx vs. 3m mdx + ABX	0,0094
			3m mdx + ABX vs. 3m GFmdx	0,0213
		IIB Fibers	3m C57Bl vs. 3m mdx + ABX	0,0094
			3m mdx vs. 3m mdx + ABX	<0,0001
			3m mdx vs. 3m GFmdx	0,0844
		IIX Fibers	3m mdx vs. 3m mdx + ABX	0,0143
		I Fibers	3m C57Bl vs. 3m GFmdx	0,011
			3m mdx + ABX vs. 3m GFmdx	0,0293
Figure 6I	Myosin isoform area	IIA Fibers	3mC57Bl vs. 3m mdx + ABX	0,0005
			3mC57Bl vs. 3m GFmdx	<0,0001
			3m mdx vs. 3m mdx + ABX	0,0032
			3m mdx vs. 3m GFmdx	<0,0001
			3m mdx + ABX vs. 3m GFmdx	<0,0001
		IIB Fibers	3mC57Bl vs. 3m GFmdx	<0,0001
			3m mdx vs. 3m GFmdx	0,0074
			3m mdx + ABX vs. 3m GFmdx	0,0001

		IIX Fibers	3m mdx vs. 3m mdx + ABX	<0,0001
			3m mdx vs. 3mC57Bl	0,0001
			3m mdx + ABX vs. 3m GFmdx	<0,0001
			3m mdx + ABX vs. 3mC57Bl	0,0049
			3m GFmdx vs. 3mC57Bl	0,0002
**Figure 6J**	SDH+ myofiber		3m C57Bl vs. mdx 3m+ABX SDH+	0,0009
			GFM3m SDH+ vs. mdx 3m+ABX SDH+	0,0001
**Figure 6K**	Tetanic force		3m mdx vs. 3m mdx + ABX	<0,0001
			3m mdx vs. 3m GFmdx	<0,0001
			3m mdx + ABX vs. 3m GFmdx	<0,0001
**Figure 6L**	Serum analysis	ALT	3m C57Bl vs. 3m mdx	0,0019
			3m C57Bl vs. 3m GFmdx	0,0001
			3m mdx + ABX vs. 3m GFmdx	0,0201
		AST	3m C57Bl vs. 3m GFMmdx	<0,0001
			3m mdx vs. 3m GFMmdx	0,0472
		CPK	3m C57Bl vs. 3m GFMmdx	<0,0001
			3m mdx + ABX vs. 3m GFMmdx	0,003

**Figure 7A**	FACS on intestinal cells	CD3+	3m mdx vs. 3m mdx + ABX	0,0044
		CD4+CD69+	3m mdx vs. 3m mdx + ABX	0,0058
		CD8+IFNγ+	3m mdx vs. 3m mdx + ABX	0,0028
		CD4+CD69+Ki67+	3m mdx vs. 3m mdx + ABX	0,019
		CD4+IL10+	3m C57Bl vs. 3m mdx	0,0022
		CD4+IFNγ+	3m C57Bl vs. 3m mdx	0,0214
			3m mdx vs. 3m mdx + ABX	0,0399

**Figure 7B**	FACS on spleen cells	CD155+Ly6C	3m mdx vs. 3m mdx + ABX	0,0435
		F4/80	C57Bl vs. mdx	0,0002
			C57Bl vs. ABX-mdx^{FTM_C57Bl}	0,0126
			mdx vs. ABX-mdx^{FTM_C57Bl}	0,0026
		CD4+CD44-CD62L+	C57Bl vs. mdx	0,0013
			C57Bl vs. ABX-mdx^{FTM_C57Bl}	0,0124
			mdx vs. ABX-mdx^{FTM_C57Bl}	0,0119
		CD4+CD44+CD62L-	C57Bl vs. mdx	0,0012
			mdx vs. ABX-mdx^{FTM_C57Bl}	0,0102
		CD8+CD44+CD62L-	C57Bl vs. mdx	0,0106
			C57Bl vs. ABX-mdx^{FTM_C57Bl}	0,0122
			mdx vs. ABX-mdx^{FTM_C57Bl}	0,0002
		CCR9+	C57Bl vs. mdx	<0,0001
			C57Bl vs. ABX-mdx^{FTM_C57Bl}	0,0106
			mdx vs. ABX-mdx^{FTM_C57Bl}	0,0024
		CD4+GITR+	C57Bl vs. mdx	0,0017
			mdx vs. ABX-mdx^{FTM_C57Bl}	0,0022
**Figure 7C**	FACS on muscle cells	CD4+	C57Bl vs. mdx	0,0228
			C57Bl vs. ABX-mdx^{FTM_C57Bl}	0,0019
		CD8+	C57Bl vs. mdx	0,0097
			C57Bl vs. ABX-mdx^{FTM_C57Bl}	0,0013
**Figure 7D**	Myofibers area		C57Bl vs. mdx	<0,0001
			C57Bl vs. ABX-mdx^{FTM_C57Bl}	<0,0001
			mdx vs. ABX-mdx^{FTM_C57Bl}	<0,0001
**Figure 7F**	Serum analysis	ALT	3m C57Bl vs. mdx	0,0091
			3m C57Bl vs. mdxFTM	<0,0001
			mdx vs. mdxFTM	0,001

		CPK	3m C57Bl vs. mdx	<0,0001
			3m C57Bl vs. mdxFTM	0,0008
			mdx vs. mdxFTM	<0,0001
Figure 7G	Tetanic force		c57bL vs. mdx	<0,0001
			c57bL vs. mdxFTM	0,0057
			mdx vs. mdxFTM	<0,0001
Figure 7I	Myosin isoform	IIA Fibers	mdx vs. mdxFTM	0,0027
		IIB Fibers	mdx vs. mdxFTM	0,0034
		IIX Fibers	3m C57Bl vs. mdxFTM	0,0056
		I Fibers	3m C57Bl vs. mdxFTM	0,0046
Figure 7K	SDH+ myofibers		3m C57Bl vs. mdx	0,0023
			mdx vs. mdxFTM	0,0003
Figure 7M	Endotehlial counts	CD31+ cells	c57bL vs. mdx	0,0333
			c57bL vs. mdxFTM	0,0333
			mdx vs. mdxFTM	<0,0001
		Isolectin+ cells	c57bL vs. mdx	0,0069
			mdx vs. mdxFTM	<0,0001
		αSMA+ cells	c57bL vs. mdxFTM	0,0057
			mdx vs. mdxFTM	<0,0001

Figure EV2A	Western Blot	IL6	c57bl vs. mdx+ABX	0,0157
			c57bl vs. GFmdx	0,0095
			mdx vs. mdx+ABX	0,0496
		MMP9	c57bl vs. mdx+ABX	0,0279
			c57bl vs. GFmdx	0,0205
		NF-kB	c57bl vs. mdx	0,0433

			mdx vs. mdx+ABX	0,0168
			mdx vs. GFmdx	0,0249
		RelB	c57bl vs. mdx	0,0442
			mdx vs. GFmdx	0,0191
**Figure EV2B**	Western Blot	IRS-1	c57bl vs. mdx	0,0217
			c57bl vs. GFmdx	0,0341
		IGF-2R	c57bl vs. GFmdx	0,0258
**Figure EV2C**	Western Blot	GHR	c57bl vs. mdx	0,0084
			c57bl vs. GFmdx	0,0015
			mdx vs. GFmdx	0,0009
**Figure EV2C**	RT-qPCR	ldh	3m C57Bl vs. 3m mdx	0,0434
		pdh	3m C57Bl vs. 3m mdx + ABX	0,0183
			3m mdx vs. 3m mdx + ABX	0,0024
		pdk4	3m C57Bl vs. 3m GFmdx	0,0317
			3m mdx + ABX vs. 3m GFmdx	<0,0001
**Figure EV2E**	Western Blot	HDAC1	c57bl vs. GFmdx	0,0011
			mdx vs. GFmdx	0,0002
			mdx+ABX vs. GFmdx	<0,0001
		AKT 1/2/3	c57bl vs. mdx	0,0006
			c57bl vs. GFmdx	0,006
			mdx vs. GFmdx	0,0144
		PPAR-γ	c57bl vs. GFmdx	0,0133
		AMPK1α	mdx vs. mdx+ABX	0,0005
			mdx vs. GFmdx	0,022
		pSMAD2/3/SMAD3	mdx vs. GFmdx	0,0047
		PGC-1α	c57bl vs. mdx	0,0082

			c57bl vs. mdx+ABX	0,022
			mdx vs. GFmdx	0,0214
**Figure EV2F**	Western Blot	TOMM20	c57bl vs. mdx	0,0001
			mdx vs. mdx+ABX	0,0098
		COXIV	c57bl vs. GFmdx	0,0294
**Figure EV2G**	RT-qPCR	cox5a	3m mdx + ABX vs. 3m GFmdx	0,0133
		cox7b	3m C57Bl vs. 3m mdx + ABX	0,0215
**Figure EV2H**	Western Blot	TRPC1	c57bl vs. mdx	0,0035
			c57bl vs. mdx+ABX	0,0036
			c57bl vs. GFmdx	0,0253
		SIRT-1	mdx vs. GFmdx	0,0151
**Figure EV2I**	RT-qPCR	casq	3m C57Bl vs. 3m GFmdx	0,0007
			3m mdx vs. 3m GFmdx	0,0231
**Figure EV2J**	Western Blot	P62	3m C57Bl vs. 3m mdx	0,0412
		FOXO1	c57bl vs. mdx	0,0154
			mdx vs. GFmdx	0,0281
**Figure EV2K**	RT-qPCR	chra	3m C57Bl vs. 3m mdx	0,0169
		chrd	3m C57Bl vs. 3m GFmdx	0,0062
			3m mdx vs. 3m GFmdx	0,0001
			3m mdx + ABX vs. 3m GFmdx	0,0328

**Figure EV4A**	FACS on intestinal cells	CD8+CD69+ cells	3m C57Bl vs. 3m mdx	0,042
		CD4+IL10+IL17+ cells	3m C57Bl vs. ABX-mdxFTM_C57Bl	0,0077
		CD4+CD62L+ cells	3m C57Bl vs. 3m mdx	0,0249
			3m C57Bl vs. ABX-mdxFTM_C57Bl	0,0198

Figure EV4B	FACS on spleen cells	Ly6G+ live cells	C57Bl vs. mdx	<0,0001
			C57Bl vs. ABX-mdx^{FTM_C57Bl}	0,0214
		CD11b+ live cells	C57Bl vs. mdx	0,0047
			C57Bl vs. ABX-mdx^{FTM_C57Bl}	0,0175
		CD44+CD62L+CD4+	C57Bl vs. mdx	0,0193
			C57Bl vs. ABX-mdx^{FTM_C57Bl}	0,0001
Figure EV4C	FACS on muscle cells	Granulocytes	C57Bl vs. mdx	0,0497
		Monocytes	C57Bl vs. ABX-mdx^{FTM_C57Bl}	0,0173
		CD115-Ly6C+ cells	C57Bl vs. mdx	0,0399
			C57Bl vs. ABX-mdx^{FTM_C57Bl}	0,0112
		Macrophages	C57Bl vs. mdx	0,0241
			C57Bl vs. ABX-mdx^{FTM_C57Bl}	0,0151
Figure EV4D	Serum analysis	AST	3m C57Bl vs. mdx	0,0008
			3m C57Bl vs. mdxFTM	<0,0001
		GLUC-3	3m C57Bl vs. mdx	0,0167
			3m C57Bl vs. mdxFTM	0,0009
