# Supplementary material for: Microbiota dysbiosis influences immune system and muscle pathophysiology of dystrophin deficient mice
Source: EMBO Mol Med. 2026 Jun 3;18(7):2979–3008. doi: 10.1038/s44321-026-00445-1 (PMC13365508; doi:10.1038/s44321-026-00445-1)
Supplement: Supplementary file 15 — Expanded View Figures [file 44321_2026_445_MOESM15_ESM.pdf]

Expanded View Figures

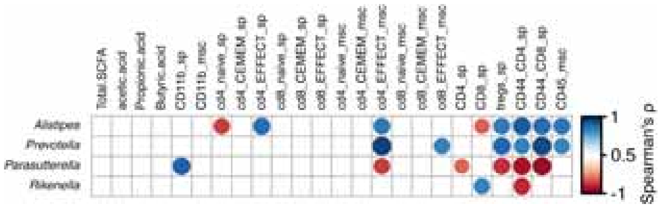

Figure EV1. Correlation between bacterial genera and immunity.

Heatmap of Spearman's rho correlations between the relative abundance of the most represented bacterial genera (with relative abundance > 0.1%) in the gut microbiota of 3 m mdx animals ($n = 3$–8) with the indicated metabolites and immunological parameters. The significant correlations with FDR-corrected P value < 0.1 are indicated with bubbles. Spearman correlation plots for the significant correlations between Prevotella and the indicated immunological parameters are also shown. sp spleen-derived, msc muscle-derived, CEMEM central memory cells, EFFECT effector cells. Source data are available online for this figure.

© The Author(s)

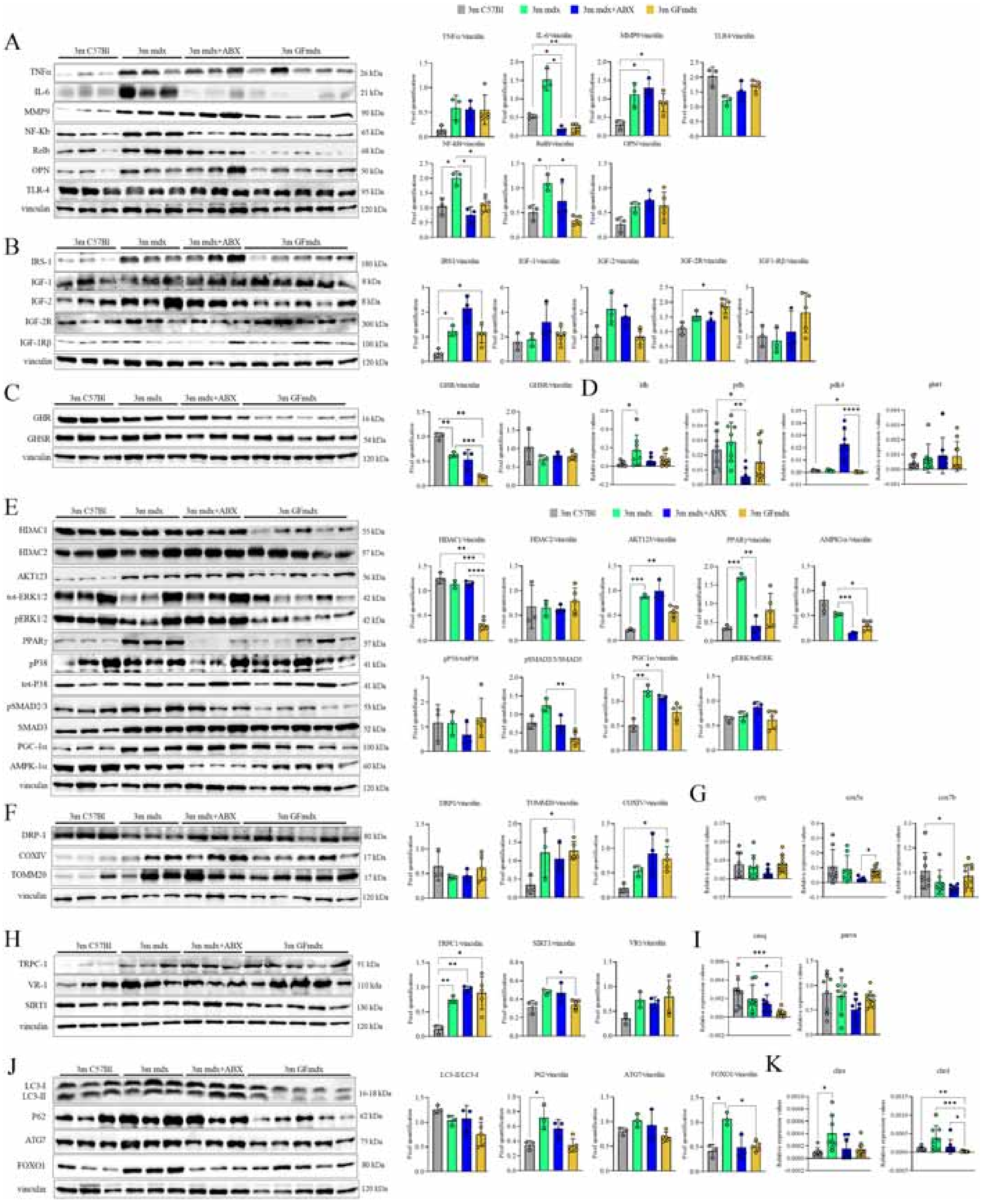

Figure EV2. Gene and protein expression in muscles from 3 m mdx, mdx + ABX, and GFmdx.

Cropped images of representative WB and RT-qPCR analysis of TA muscle of 3 m mdx ($n = 3/4$), 3 m mdx+ABX ($n = 3/4$) and 3 m GFmdx ($n = 5$) showing the expression of the proteins specifically involved in inflammation/fibrosis (**A**), skeletal muscle metabolism (**B–E**), mitochondrial biogenesis (**F**, **G**), calcium conducting channels (**H**, **I**), autophagy (**J**) and Nicotinic acetylcholine receptors (**K**). Densitometric data were normalized on vinculin and expressed as mean ± SD. Data are presented as mean ± SD (*$P < 0.05$, **$P < 0.01$, ***$P < 0.001$; ****$P < 0.0001$, ordinary one-way ANOVA, Tukey multiple comparison test for WB and nonparametric test followed by Kruskal–Wallis test for RT-qPCR). Source data are available online for this figure.

© The Author(s)

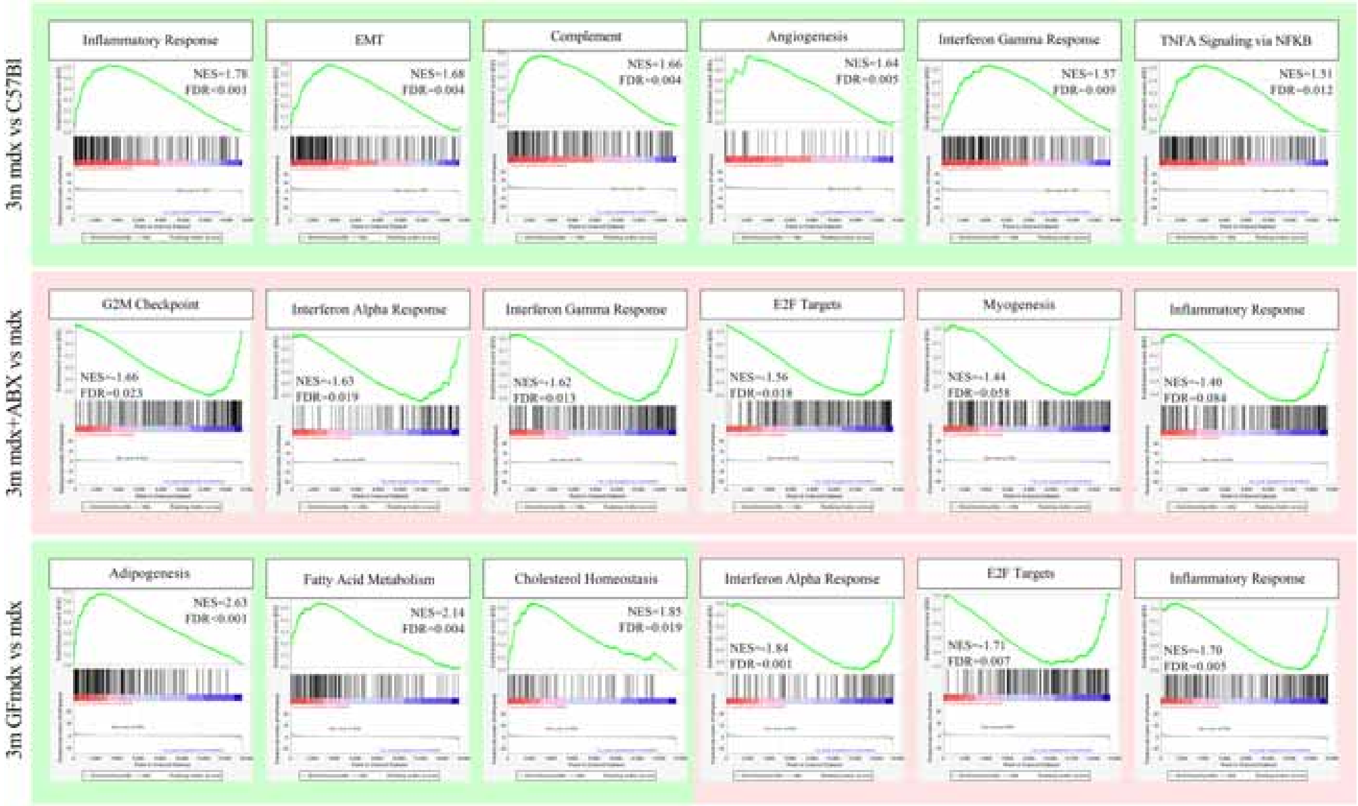

Figure EV3. Gene set enrichment analysis (GSEA) of 3 m C57bl, mdx, mdx + ABX, and GFmdx mice RNA sequencing data.

The annotated dataset "Hallmark" collection by the Molecular Signatures Database (MSigDB) was used as a reference. A green background refers to positive Normalized Enrichment Score (NES) (enrichment in positive phenotype, or upregulation); a red background refers to negative NES (enrichment in negative phenotype, or downregulation). FDR: False discovery rate. Genes involved in inflammatory response, epithelial-to-mesenchymal transition (EMT), complement activity, angiogenesis, and interferon-γ response are upregulated in 3 m mdx ($n = 3$) vs age-matched C57Bl ($n = 3$) mice (top lane). Genes involved in G2M checkpoint transition, interferon-α and -γ response, E2F transcriptional activity, and myogenesis are downregulated in 3 m mdx+ABX ($n = 3$) vs age-matched mdx ($n = 3$) mice (mid lane). Genes involved in adipogenesis, fatty acid metabolism, and cholesterol homeostasis are upregulated in 3 m GFmdx ($n = 3$) vs age-matched mdx ($n = 3$) mice; conversely, genes involved in interferon alpha response, E2F transcriptional activity, and inflammatory response are downregulated in the 3 m GFmdx vs age-matched mdx mice (bottom lane). Source data are available online for this figure.

 © The Author(s)

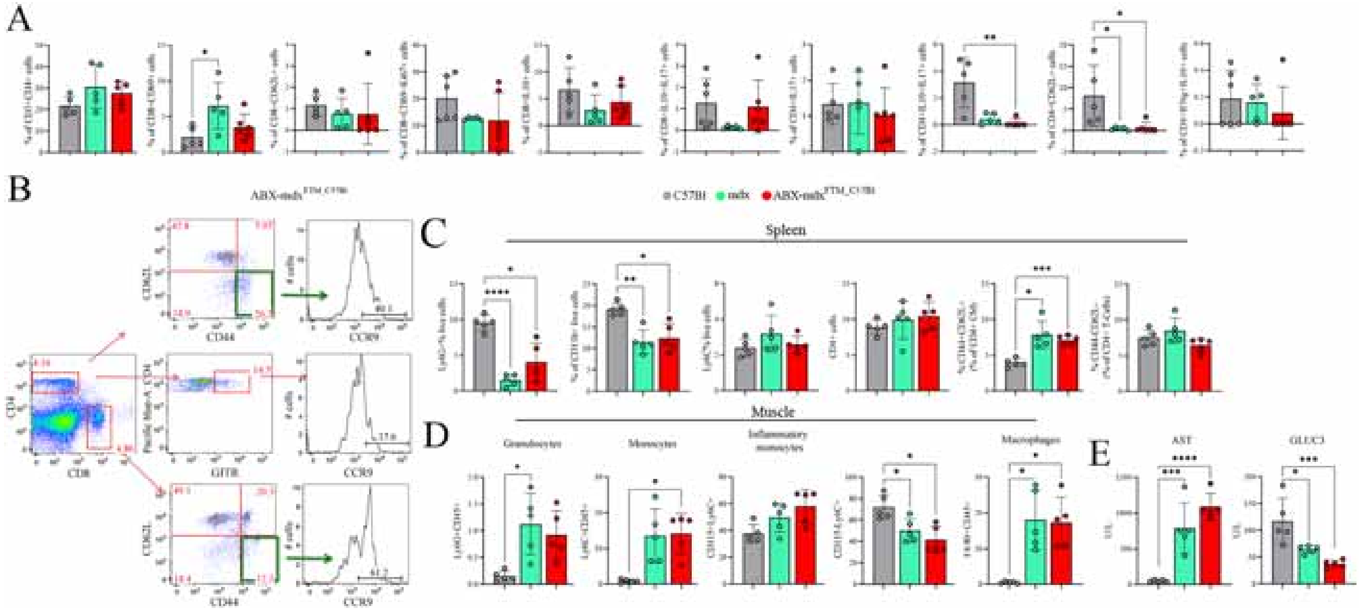

Figure EV4. Effects of dysbiotic microbiota of mdx on intestinal, spleen, and muscle inflammation.

(**A**) FACS analysis of colon lamina propria of mdx ($n = 5$) and ABX-mdx^{FMT_C57Bl} ($n = 5/6$) for quantification of T-cell subsets. (**B**) Representative plots of FACS analysis for the expression of CCR9 in ABX-mdx^{FMT_C57Bl} and ABX-C57Bl^{FMT_mdx} are depicted. The numbers within the panels indicate the percentage of each population of live cells. Each analysis included at least 5–10 × 10^4 events for each gate. FACS analysis of T cells of spleen (**C**) and granulocyte, monocyte, and macrophage of muscle (**D**) tissues from mdx ($n = 5$) and ABX-mdx^{FMT_C57Bl} ($n = 5/6$). (**E**) Serum levels of AST and GLUC3. Data are presented as mean ± SD ($^*P < 0.05$; $^{**}P < 0.01$; $^{****}P < 0.0001$, ordinary one-way ANOVA, Tukey's multiple comparison test). Source data are available online for this figure.

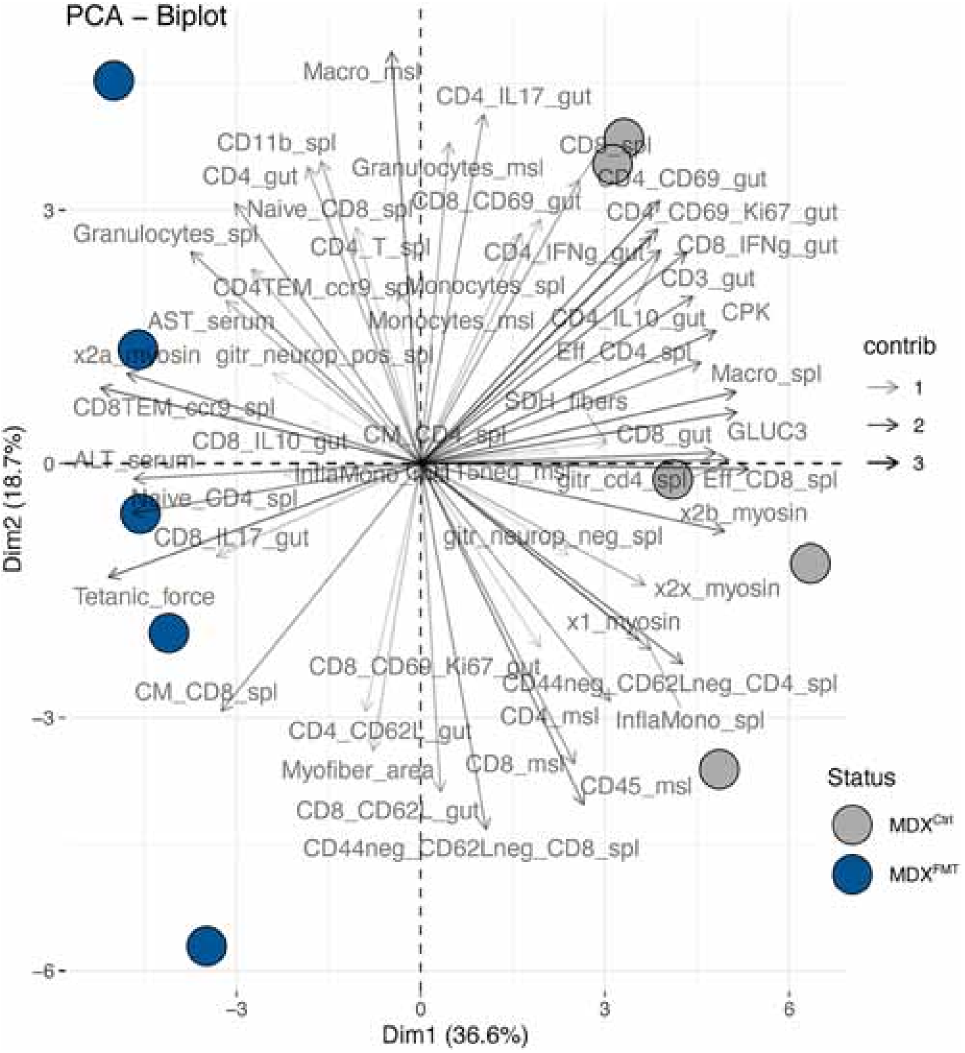

Figure EV5. Principal component analysis (PCA) biplot of samples and analyzed variables from spleen, gut, and muscle.

The biplot shows the PCA scores of the explanatory variables as vectors and samples colored according to treatment and genetic backgrounds. The color intensity of the vectors (lines) shows the strength of their contribution to each PC. Vectors pointing in similar directions indicate positively correlated variables, vectors pointing in opposite directions indicate negatively correlated variables.

 © The Author(s)
